# How to Best Protect People With Diabetes From the Impact of SARS-CoV-2: Report of the International COVID-19 and Diabetes Summit

**DOI:** 10.1177/1932296820978399

**Published:** 2021-01-21

**Authors:** Jennifer Y. Zhang, Trisha Shang, David Ahn, Kong Chen, Gerard Coté, Juan Espinoza, Carlos E. Mendez, Elias K. Spanakis, Bithika Thompson, Amisha Wallia, Lauren E. Wisk, David Kerr, David C. Klonoff

**Affiliations:** 1 Diabetes Technology Society, Burlingame, CA, USA; 2 Mary & Dick Allen Diabetes Center, Hoag Memorial Hospital Presbyterian, Newport Beach, CA, USA; 3 National Institutes of Health, National Institute of Diabetes and Digestive and Kidney Diseases, Bethesda, MD, USA; 4 Texas A&M Engineering Experiment Station Center for Remote Health Technologies and Systems, Department of Biomedical Engineering, Texas A&M University, College Station, TX, USA; 5 Children’s Hospital Los Angeles, University of Southern California, Los Angeles, CA, USA; 6 Medical College of Wisconsin, Milwaukee, WI, USA; 7 School of Medicine, University of Maryland, Baltimore, MD, USA; 8 Division of Endocrinology, Baltimore Veterans Affairs Medical Center, Baltimore, MD, USA; 9 Mayo Clinic Arizona, Scottsdale, AZ, USA; 10 Feinberg School of Medicine, Northwestern University, Chicago, IL, USA; 11 David Geffen School of Medicine, University of California, Los Angeles, CA, USA; 12 Sansum Diabetes Research Institute, Santa Barbara, CA, USA; 13 Mills-Peninsula Medical Center, San Mateo, CA, USA

**Keywords:** COVID-19, diabetes, digital health, pandemic, telehealth

## Abstract

The coronavirus disease 2019 (COVID-19) pandemic caused by the severe acute respiratory syndrome coronavirus 2 (SARS-CoV-2) virus has rapidly involved the entire world and exposed the pressing need for collaboration between public health and other stakeholders from the clinical, scientific, regulatory, pharmaceutical, and medical device and technology communities. To discuss how to best protect people with diabetes from serious outcomes from COVID-19, Diabetes Technology Society, in collaboration with Sansum Diabetes Research Institute, hosted the “International COVID-19 and Diabetes Virtual Summit” on August 26-27, 2020. This unique, unprecedented real-time conference brought together physicians, scientists, government officials, regulatory experts, industry representatives, and people with diabetes from six continents to review and analyze relationships between COVID-19 and diabetes. Over 800 attendees logged in. The summit consisted of five sessions: (I) Keynotes, (II) Preparedness, (III) Response, (IV) Recovery, and (V) Surveillance; eight parts: (A) Background, (B) Resilience, (C) Outpatient Care, (D) Inpatient Care, (E) Resources, (F) High-Risk Groups, (G) Regulation, and (H) The Future; and 24 sections: (1) Historic Pandemics and Impact on Society, (2) Pathophysiology/Risk Factors for COVID-19, (3) Social Determinants of COVID-19, (4) Preparing for the Future, (5) Medications and Vaccines, (6) Psychology of Patients and Caregivers, (7) Outpatient Treatment of Diabetes Mellitus and Non-Pharmacologic Intervention, (8) Technology and Telehealth for Diabetes Outpatients, (9) Technology for Inpatients, (10) Management of Diabetes Inpatients with COVID-19, (11) Ethics, (12) Accuracy of Diagnostic Tests, (13) Children, (14) Pregnancy, (15) Economics of Care for COVID-19, (16) Role of Industry, (17) Protection of Healthcare Workers, (18) People with Diabetes, (19) International Responses to COVID-19, (20) Government Policy, (21) Regulation of Tests and Treatments, (22) Digital Health Technology, (23) Big Data Statistics, and 24) Patient Surveillance and Privacy. The two keynote speeches were entitled (1) COVID-19 and Diabetes—Meeting the Challenge and (2) Knowledge Gaps and Research Opportunities for Diabetes and COVID-19. While there was an emphasis on diabetes and its interactions with COVID-19, the panelists also discussed the COVID-19 pandemic in general. The meeting generated many novel ideas for collaboration between experts in medicine, science, government, and industry to develop new technologies and disease treatment paradigms to fight this global pandemic.

## Introduction

**Figure 1.**
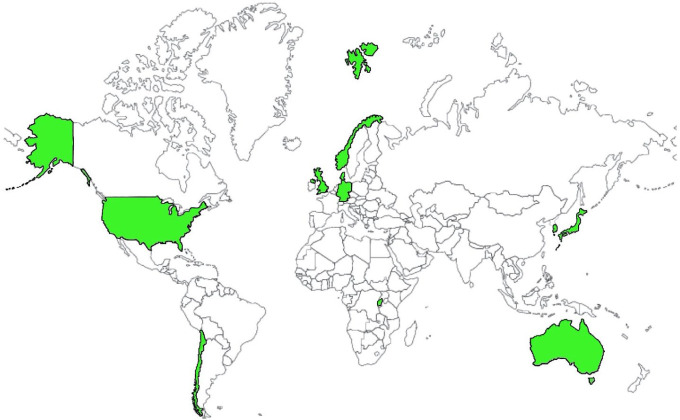
A map of the countries (in green) where Summit participants were based. Participants were from Africa, Asia, Australia, Europe, North America, and South America and were based in Australia, Chile, Denmark, Germany, Japan, Norway, Rwanda, South Korea, the United Kingdom (UK), and the United States of America (USA). Figure adapted from “Planisphère (Projection Mercator), 2015.”^3^

The COVID-19 pandemic has been classified as a global health emergency by the World Health Organization (WHO).^1^ People with diabetes are particularly susceptible to negative outcomes when infected by severe acute respiratory syndrome coronavirus 2 (SARS-CoV-2).^2^ To discuss how to best protect people with diabetes from serious outcomes from COVID-19, Diabetes Technology Society, in collaboration with Sansum Diabetes Research Institute, hosted the “International COVID-19 and Diabetes Virtual Summit” on August 26-27, 2020, which featured 79 speakers and eight moderators. Participants were from Africa, Asia, Australia, Europe, North America, and South America and were based in Australia, Chile, Denmark, Germany, Japan, Norway, Rwanda, South Korea, the United Kingdom (UK), and the United States of America (USA) (Figure 1). The participants were experts in COVID-19 and/or diabetes. The meeting was divided into five sessions, which included keynote presentations as one of the sessions, plus four additional sessions, which each contained two parts. The meeting’s eight parts were divided into 24 sections, each consisting of presentations by a set of experts and a panel discussion. This meeting report summarizes the Key Points of each speaker and the major themes discussed by the panels in each of the 24 sections of the meeting.

Many speakers pointed out a very recent trend in healthcare, precipitated by the COVID-19 pandemic, for patients and the healthcare system to interact by way of electronic communication tools. In this report, we used the following definitions of telehealth and telemedicine given by the United States Health Resources Services Administration: (1) telehealth is the use of electronic information and telecommunications technologies to support clinical services as well as remote non-clinical services, such as provider training, administrative meetings, and continuing medical education; and (2) telemedicine is a part of telehealth and refers to remote clinical services.^4^

## Session I: Keynote Speeches

***Day 1*: Robert A. Gabbay, MD, PhD**

American Diabetes Association, Arlington, Virginia, USA

**Key Points:**

- The COVID-19 pandemic has fundamentally changed how healthcare is delivered.
- Telehealth and the sharing of data have become indispensable tools for managing people living with diabetes during the time of COVID-19.
- Some of the significant changes in healthcare delivery brought upon by the COVID-19 pandemic are likely to remain even after the pandemic is under control.

### Summary

While the COVID-19 pandemic has fundamentally changed healthcare delivery, at the same time, health disparities have been exposed. COVID-19 has had a disproportionate impact on people of color and has shown profound economic challenges, including pushing low-income and self-employed people with diabetes to self-ration supplies in order to reduce costs. On a more positive note, the pandemic has served as an accelerant for innovation with telehealth and the sharing of data, which have become indispensable tools to manage diabetes during the time of COVID-19. The American Diabetes Association (ADA) has rapidly assembled a robust response to the COVID-19 pandemic, including: (1) healthcare education with a series of webinars from a core leadership team of experts that shared early learnings on inpatient and outpatient care, team-based approaches, patient empowerment, and mental health; (2) new research funding (see Table 1); and (3) patient resources. Some of the important questions that need to be answered include: (1) the role of inpatient glycemic control for hospitalized patients infected with SARS-CoV-2; (2) the impact of corticosteroids in patients with diabetes or hyperglycemia; and (3) the interplay between kidney disease and COVID-19 in patients with diabetes. The potential that COVID-19 has to increase the risk of diabetes after recovery and which specific diabetes medications may have a role in COVID-19 infection treatment also require further research.

**Table 1.** The ADA’s Research Response to COVID-19.

May 11, 2020	The ADA launched a request for application to fund ten, one-year research grants focused on Diabetes and COVID-19.
June 5, 2020	An ad hoc review committee was formed and reviewed 212 proposals.
July 1, 2020	Ten grants were funded.

*Note*. Table provided by Robert A. Gabbay, MD, PhD, Chief Scientific and Medical Officer of the ADA.

Abbreviations: ADA: American Diabetes Association; COVID-19: coronavirus disease 2019.

***Day 2*: William Cefalu, MD**

National Institutes of Health, National Institute of Diabetes and Digestive and Kidney Diseases, Bethesda, Maryland, USA

**Key Points:**

- Individuals with metabolic conditions such as diabetes mellitus (DM) and/or obesity have increased risk of morbidity and mortality from COVID-19 infection.
- The mechanism by which SARS-CoV-2 infects organs and contributes to increased risk (eg, diabetes and other metabolic diseases, obesity) is poorly defined. Individual susceptibility to infection and acute and long-term sequelae of COVID-19 are largely unknown.
- Given the clinical significance of the extra-pulmonary manifestations of COVID-19, including abnormalities of glucose metabolism, research is clearly needed to better understand the heterogeneity of individual response to SARS-CoV-2 infection.
- The National Institutes for Health - National Institute of Diabetes and Digestive and Kidney Diseases (NIH-NIDDK) has solicited new research for rapid translation and impact to address COVID-19 and metabolic diseases. The NIH-NIDDK also aims to delineate and address mechanisms by which people with diseases in the mission of NIDDK have poor outcomes from SARS-CoV-2 infection, including variable susceptibility, altered course of disease, morbidity, and mortality.

### Summary

**Figure 2.**
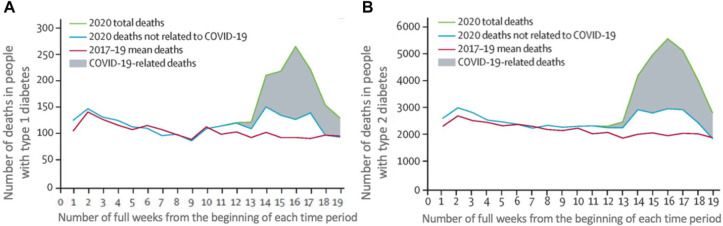
The mean weekly mortality rate during 2017-2019 (prior to the COVID-19 pandemic), compared to the weekly mortality rate during 2020 (the first year of the COVID-19 pandemic), during the first 19 weeks of these two time periods in the UK for people with T1D and T2D.^7^ The x axis shows the number of full weeks following the beginning of the two time periods. Colored lines indicate mean total weekly death rates during the period 2017-2019 (red line), total weekly death rates in 2020 (green line), and weekly death rates not related to COVID-19 in 2020 (blue line). The gray shadow represents deaths related to COVID-19 during 2020. (a) The weekly mortality rate (on the y axis) of people with T1D. (b) The weekly mortality rate (on the y axis) of people with T2D. Abbreviations: COVID-19, coronavirus disease 2019; T1D, type 1 diabetes; T2D, type 2 diabetes.

**Figure 3.**
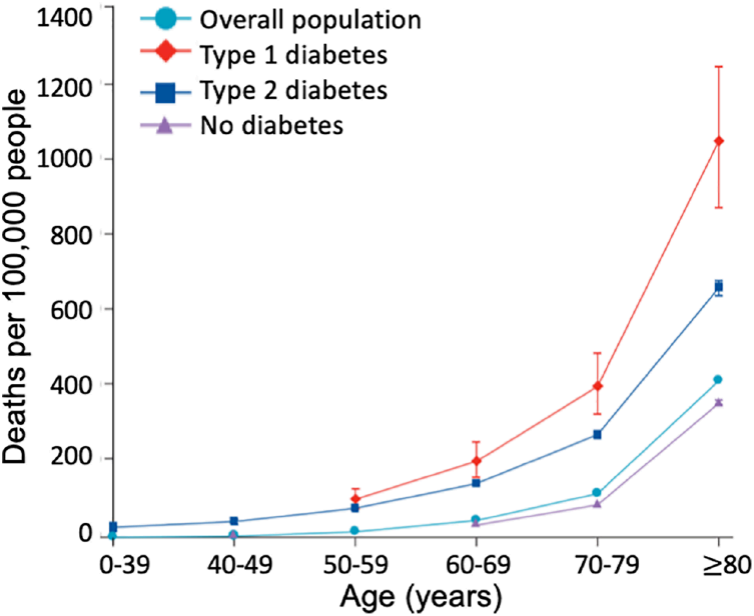
Mortality for patients in the UK with COVID-19 and diabetes, stratified according to T1D, T2D, or no diabetes.^8^ Abbreviations: COVID-19, coronavirus disease 2019; T1D, type 1 diabetes; T2D, type 2 diabetes.

**Figure 4.**
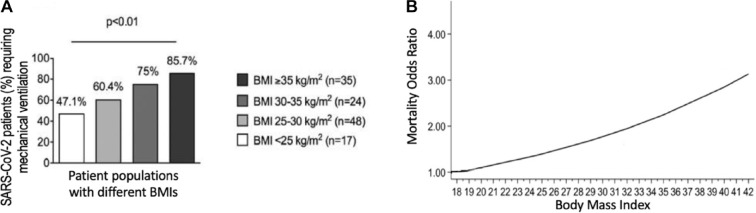
The correlation between BMI and poor clinical outcomes. (a) The correlation between BMI and requirement for mechanical ventilation for patients with COVID-19. Adapted with permission from ‘High Prevalence of Obesity in Severe Acute Respiratory Syndrome Coronavirus-2 (SARS-CoV-2) Requiring Invasive Mechanical Ventilation’.^9^ (b) The association between BMI and mortality due to COVID-19.^10^ Adapted with permission from ‘Association of Body mass index (BMI) with Critical COVID-19 and In-hospital Mortality: a dose-response meta-analysis’. Abbreviations: BMI, body mass index; COVID-19, coronavirus disease 2019.

Individuals with chronic conditions such as diabetes, cardiovascular diseases (CVDs), and chronic obstructive pulmonary disease (COPD) are at increased risk of morbidity and mortality from COVID-19. In 2020, after the start of the pandemic, the number of deaths exceeded the mean number of deaths for the corresponding weeks in the preceding three years in people with type 1 diabetes (T1D) and type 2 diabetes (T2D), as seen in Figures 2(a) and (b), which present data from the UK. Mortality for patients with COVID-19 and diabetes increases substantially with age, as seen in Figure 3, which also presents data from the UK. The presence of poor glycemic control both in patients with T1D and T2D is associated with higher mortality from COVID-19.^5^ Obesity has been identified as an important risk factor for morbidity, and there is a correlation between body mass index (BMI) and poor clinical outcomes, such as need for mechanical ventilation or death, as seen in Figures 4(a) and (b). Additionally, age, male sex, and excess adiposity have been influencing factors in the cytokine storm seen during SARS-CoV-2 infection. Moreover, SARS-CoV-2 infection seems to disproportionally affect racial minorities.^6^ These findings have created areas of research interest that could help clinicians better manage at-risk or affected patients. The NIH-NIDDK solicited new research for rapid translation and impact to address COVID-19 and metabolic diseases and to delineate and address mechanisms by which people with diseases have poor outcomes from SARS-CoV-2 infection. These mechanisms might include variable susceptibility, altered course of disease, and differences in morbidity and mortality. As such, the collection of bio-samples to better understand pathogenesis and association with underlying conditions as well as clinical trials to determine differences in subject characteristics, therapies, and impact on outcomes are of particular interest. In addition, identification of risk factors could lead to modification of therapies, novel pathogenic pathways, or pilot studies. These activities would be of the utmost value to facilitate an understanding of the natural history of the disease, its association with related conditions, and the best interventions for prevention and treatment.

## Session II: Preparedness

### Part A: Background

Moderator: Juan Espinoza, MD, FAAP

Children’s Hospital Los Angeles, University of Southern California, Los Angeles, California, USA

#### Section 1: Historic Pandemics and Impact on Society

**Thomas Ewing, PhD**

Virginia Tech University, Blacksburg, Virginia, USA

**Key Points:**

- Similarities between the 1918 and 2020 pandemics include the sudden appearance of an unexpected disease, rapid, and widespread increases in cases and deaths; variation in impact globally, nationally, and regionally; lack of effective treatments; sudden implementation of public health measures; and inconsistent recommendations from medical authorities.
- Differences between 1918 and 2020 include the much-improved technology for diagnosis and treatment, more awareness of the distinctions between diseases, the importance of testing as a diagnostic tool currently, an information ecosystem that is more rapid and decentralized, political polarization fostering mistrust of health policy measures, and an epidemic now anticipated to last for months and even years.
- Lessons to be learned from 1918 for 2020 include the importance of clear and consistent messaging about disease, recommendations for public health measures that address the need for adaptation to changing circumstances, more effective political leadership to implement and sustain difficult measures, and awareness of the differential impact of epidemic disease with and across societies.

**Christopher McKnight Nichols, PhD**

Oregon State University, Corvallis, Oregon, USA

**Key Points:**

- During the 1918 pandemic, there were cancellations and postponements of large events, gatherings, other activities, school closures, anti-“crowding” measures, as well as efforts to take more precautions, including what we would now call “social distancing,” hand hygiene, masks, and related efforts. These measures worked to slow spread and limit suffering, disease, and death.
- Most Western nations were involved in the First World War. During this time, there were attempts to control information, minimize risks, and hide real data about infection and mortality. The media and government agencies in the USA explicitly sought to hide information related to the pandemic to enhance the war effort.
- Honest information is key with early, continued action led by data. As public health officials put it during the deadly second wave in the fall of 1918: “it is easier to prevent than cure.”

#### Summary of Panel

The 1918 influenza pandemic, caused by an H1N1 virus, affected approximately 500 million people worldwide, with an estimated 50 million deaths globally and 675,000 deaths in the USA.^11^ There are several similarities between 1918 influenza pandemic and the 2020 COVID-19 pandemic, including the sudden appearance of an unexpected disease, rapid and widespread increases in cases and deaths, lack of effective pharmacologic interventions, sudden implementation of public health measures with significant regional variability, and inconsistent recommendations from medical authorities. Public health measures focused on individual and organizational behavior modifications. Anti-crowding measures (cancellations and postponements of large events, gatherings, other activities, school closures, etc.), physical distancing (also called social distancing), hand hygiene, and masks were all measures that were recommended in 1918, and when implemented, helped to slow the spread of disease and limit suffering, disease, and death.^12^ It is also worth noting that the 1918 influenza pandemic occurred while many Western nations were involved in World War I. Governments around the world tried to control information by obscuring infection and mortality rates and pushed nationalistic messaging and activities. In the USA, public media contributed to these efforts along with government agencies like the Woodrow Wilson Administration’s Committee on Public Information, which explicitly sought to hide infections and minimize risks to enhance the war effort.

Despite these similarities, there are several key differences between the 1918 and 2020 pandemics. These include much-improved technology for diagnosis and treatment, more awareness of the distinctions between diseases, and recognition of the importance of testing as a diagnostic tool. In addition, we now have an information ecosystem that is more rapid and decentralized. Political polarization fostering mistrust of health policy measures, while not new as a phenomenon, seems to have a larger impact in 2020 than 1918. Finally, while the 1918 influenza epidemic resolved by 1919, the current epidemic is anticipated to last for months and even years, according to some estimates.

There are several key lessons we can learn from the 1918 influenza epidemic. First, clear and consistent messaging about the disease from public health officials is critical. Second, public health measures need to adapt to changing circumstances as we learn more about the disease, or as the pandemic evolves. Third, effective political leadership is crucial to sustaining difficult societal measures. Finally, it is important to be aware of the differential impact of epidemic disease across society, particularly the disproportionate impact on marginalized and underserved communities.

#### Section 2: Pathophysiology/Risk Factors for COVID-19

**George Rutherford III, MD, MA**

University of California, San Francisco, San Francisco, California, USA

**Key Points:**

- Diabetes is one of the preexisting conditions associated with increased severity of SARS-CoV-2 infection.
- In adolescents and children, obesity is a major predisposing condition.
- We should anticipate a third wave of infection this fall, superimposed on the current epidemic, which will involve middle school, high school and college students.

**Mercedes Carnethon, PhD**

Northwestern University, Chicago, Illinois, USA

**Key Points:**

- The reason that diabetes confers elevated risk for adverse outcomes from COVID-19 may be because of the adverse social determinants of health (SDoH) that interfere with diabetes management.
- Comorbid obesity interferes with best practices for prone intubation in severe COVID-19.
- Persons across the age range with T2D should be prioritized for vaccination, and tailored messaging to these groups needs to be developed, given a historical hesitancy for vaccine uptake.

**Simin Liu, MD, ScD, MPH, MS**

Brown University, Providence, Rhode Island, USA

**Key Points:**

- Patients with diabetes and COVID-19 are at increased risk for hospitalization, intensive care unit (ICU) admission, mortality, or ventilation.
- Potential genomic and biological mechanisms with implications for sex differences in the infectivity and severity of COVID-19 in the cardiometabolic space need to be explored.
- Further research needs to be conducted to understand risk factors and biomarkers for patients who have COVID-19 and a preexisting condition. Interactions between COVID-19 and susceptibility, as well as drugs or therapies that might affect immunometabolism by age and sex, must be identified and characterized.

**Darin Olson, MD, PhD**

Emory University, Atlanta, Georgia, USA

**Key Points:**

- The data are changing before our eyes, while we focus on hospitalizations and mortality with current reports.
- An approximate monthly timeline of new findings demonstrates that metabolic disease is related to COVID-19 epidemiology. There was increased mortality with DM and associated conditions originally reported in China—with additional increased mortality later reported in Italy and expanded observations on the effects of racial and ethnic disparity combined with diabetes and obesity comorbidity reported later as well.
- More rigorous epidemiological studies will continue to define the relationships between diabetes and associated bio-psycho-social conditions with COVID-19.

#### Summary of Panel

**Figure 5.**
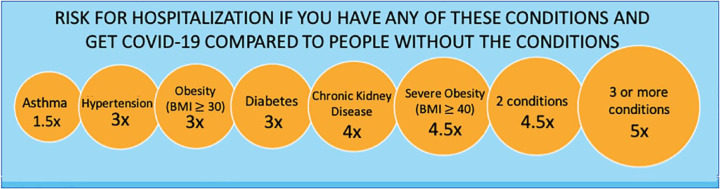
Conditions that increase the risk of hospitalization for COVID-19 patients. According to the Centers for Disease Control and Prevention (CDC) COVID-19 Digital Resources website as of August 27, 2020,^15^ there is increased risk for hospitalization from contracting COVID-19 for individuals with various conditions, including (1) asthma, (2) hypertension, (3) obesity, (4) diabetes, (5) chronic kidney disease, (6) severe obesity, (7) two conditions, and (8) three or more conditions. These conditions consist of the previously listed first six conditions (but not hypertension), and three additional conditions, including coronary artery disease, history of stroke, and COPD. Abbreviations: COPD, chronic obstructive pulmonary disease; COVID-19, coronavirus disease 2019.

Diabetes confers a three-fold increase in risk of severe outcomes (defined as hospitalization, ICU admission, intubation, or death) compared to individuals without the disease.^13^ Many of the common comorbidities of T2D, including hypertension (HTN), obesity, coronary artery disease, and chronic kidney disease, further compound the risk of severe outcomes, up to five-fold greater than the general population. Conditions that increase the risk of hospitalization for COVID-19 patients are shown in Figure 5. Some studies suggest that aggressive control of diabetes may result in better outcomes.^14^ Although individuals with diabetes are more likely to contract certain infections because of immune dysregulation, diabetes does not appear to confer a greater risk for contracting COVID-19, though there are insufficient data to definitively rule this out. Additional social factors, which are yet to be defined, appear to place persons with diabetes at increased risk of exposure and infection.

There are several other identified risk factors for COVID-19 that are also commonly associated with diabetes: older age, overweight and obesity, male sex, nonwhite race/ethnicity (specifically, Native American, Black, South Asian, and Latinx), and two or more chronic conditions. Obesity in particular seems to be a major factor (85% of individuals with T2D have obesity). Obesity may be linked to increased disease severity through several mechanisms, including underlying impairment of the cardiovascular, respiratory, metabolic and thrombotic pathways, a pro-inflammatory or dysregulated immune response, and potentially increased viral shedding.^16^ Obese adults may also have more difficulty with effective treatment such as prone positioning, which can delay intubation and improve outcomes once ventilated.^17^

Biologically, estrogen may have protective effects and may explain, at least in part, some of the gender-based differences.^18^ The immune-stimulatory genes encoded from the two X-chromosomes in women versus one X- and one Y-chromosome in men also influence the gender-based difference. Angiotensin-converting enzyme 2 (ACE2) and transmembrane protease serine type 2 (TMPRSS2) have been implicated as key molecules in SARS-CoV-2 infection.^19,20^ The relative overexpression of ACE2 and TMPRSS2 in men may contribute to their increased viral load and decreased viral clearance capacity. Men, compared to women, are characterized by an increased intrinsic propensity to meta-inflammation leading to cytokine storm. These hypotheses can be tested in further epidemiological observation. Careful targeting of the renin-angiotensin system axis and cytokine storm may represent a strategy for improving clinical outcomes in people with diabetes infected with COVID-19.

Finally, it is worth remembering that the nature of the pandemic has led to a large output of literature that is relatively low in the terms of level of evidence^21^: case series, cohort studies, and cross-sectional studies—typically, with little or no long-term follow up. There have also been methodological concerns about many of the studies published.^22^ More rigorous and systematic studies will be needed to fully understand the epidemiology and pathophysiology of COVID-19, and how it impacts people with diabetes.^23^

#### Section 3: Social Determinants of COVID-19

**Kristin Bennett, PhD**

Rensselaer Polytechnic Institute, Troy, New York, USA

**Key Points:**

- Population health studies of surveillance data provide insights into potential risk factors for COVID-19 mortality at the county level.
- Communities with greater economic and housing stress, nonwhite/immigrant populations, and worse health outcomes/access have increased COVID-19 deaths.
- Further studies are needed—the relationships between COVID-19 with asthma, suicide, and alcohol abuse are complex.

**Vickie M. Mays, PhD, MSPH**

University of California, Los Angeles, Los Angeles, California, USA

**Key Points:**

- Health conditions alone do not put individuals at risk for morbidity and mortality from COVID-19, but those conditions do put individuals at risk when they are paired with detrimental SDoH.^24^
- Efforts to prevent COVID-19 can worsen SDoH.
- Mitigating COVID-19 morbidity and mortality means mitigating unemployment, homelessness, and food insecurity.

#### Summary of Panel

SDoH are “the conditions in the environments where people are born, live, learn, work, play, worship, and age that affect a wide range of health, functioning, and quality-of-life outcomes and risks.”^25^ Factors that influence economic stability, education, social and community context, health and healthcare, and neighborhoods and the built environment are all considered SDoH (Table 2). The impact of SDoH is significant and can account for up to 80% of the influence over long-term health outcomes for a population.^26^ The differential impact of the COVID-19 pandemic across different communities in the USA has both highlighted and exacerbated the underlying health inequities driven by SDoH. Early on in the pandemic, it became obvious that Black, Latinx, and low-income communities experienced disproportionate morbidity and mortality from COVID-19.^27,28^

**Table 2.** Social Determinants of Health.

• Economic stability
— Employment
— Food insecurity
— Housing instability
— Poverty
• Education
— Early childhood education and development
— Enrollment in higher education
— High school graduation
— Language and literacy
• Social and community context
— Civic participation
— Discrimination
— Incarceration
— Social cohesion
• Health and healthcare
— Access to healthcare
— Access to primary care
— Health literacy
• Neighborhood and built environment
— Access to foods that support healthy eating patterns
— Crime and violence
— Environmental conditions
— Quality of housing
— Transportation

Table provided by Juan Espinoza, MD, FAAP, Children’s Hospital Los Angeles, University of Southern California.

By leveraging large, national datasets, it is possible to explore many of the SDoH that influence COVID-19. A recent study by Debopadhaya et al^29^ explored the association of various social determinants with COVID-19 mortality at the county level across the entire USA. They found that high rates of lacking insurance, limited English proficiency, air pollution, overcrowded housing, and lower educational attainment were all associated with increased COVID-19 mortality. Communities with large Black or African American and Latinx populations also had higher mortality. Interestingly, the study also found some protective factors without an obvious causal relationship. Communities with higher rates of suicide and excessive drinking seem to experience less COVID-19 mortality. This may be related to preexisting social isolation that, while contributing to deaths of despair, may reduce COVID-19 transmission. Communities with high asthma rates also seemed to have a lower mortality ratio. One possible explanation for this might be that the use of corticosteroids in the treatment of asthma may improve COVID-19 outcomes.^30^

SDoH results in social stressors, such as anxiety about food availability and paying rent. Income insecurity, food insecurity, and housing insecurity have all been identified as survival threats for COVID-19. From a policy perspective, we should consider unaddressed SDoH as threats for new infections, and these should be taken on not only by providers, but at the local, state, and federal policy levels. Testing policies and resources should take into consideration existing inequities of access and trust in order to meaningfully reach underserved and marginalized communities. Food and water distribution is critical, and will require collaboration across sectors, including philanthropies, churches, healthcare, and local governments. A systematic approach to addressing SDoH will help reduce the inequities we are observing in COVID-19 morbidity and mortality.

### Part B: Resilience

Moderator: Bithika Thompson, MD

Mayo Clinic Arizona, Scottsdale, Arizona, USA

#### Section 4: Preparing for the Future

**Amesh Adalja, MD**

Johns Hopkins University, Baltimore, Maryland, USA

**Key Points:**

- COVID-19 will be an endemic virus.
- High-risk individuals, even after the development of a vaccine, will still face challenges.
- Expect more pandemic and infectious disease emergencies.

#### Summary

**Figure 6.**
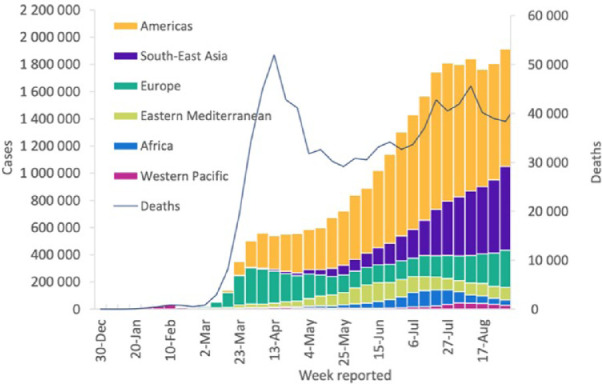
The number of COVID-19 cases and deaths from COVID-19 reported weekly in each of the six regions recognized by the WHO, according to figures from that organization. The graph covers December 30, 2019 to August 31, 2020.^31^ Abbreviation: COVID-19: coronavirus disease 2019.

COVID-19 is a respiratory virus that spreads efficiently from human to human, making it an efficient pandemic pathogen. By the time the severity of the COVID-19 pandemic was recognized, the virus had already seeded many parts of the world, changing our focus from containment to mitigation. COVID-19 cases and deaths from COVID-19 have been reported in all six of the geographic regions recognized by the WHO (Figure 6). It is predicted that moving forward, COVID-19 will establish itself as one of our seasonal coronaviruses and become endemic. People with diabetes and other individuals at highest risk of morbidity and mortality from this virus will continue to face challenges even with the development of a vaccine, because the vaccine will likely not provide sterilizing immunity. High-risk individuals will need to continually assess their risk of morbidity and mortality. We should expect more pandemics and infectious disease emergencies. In the future, we need to focus our efforts on pandemic preparedness. This should include: (1) earlier and more aggressive efforts to characterize and identify unknown diagnoses quickly, (2) better containment strategies, and (3) consistent funding for pandemic preparedness.

#### Section 5: Medications and Vaccines

**Evan Martin Bloch, MD, MS**

Johns Hopkins University, Baltimore, Maryland, USA

**Key Points:**

- Growing evidence suggests that convalescent plasma (CP) is a safe and effective treatment for COVID-19.
- There has been an unprecedented scale-up of collections, distribution, and transfusions of CP to treat COVID-19.
- Clinical trials are critically needed to confirm efficacy and optimal use; these have proved enormously challenging.

**Daniel Griffin, MD, PhD**

Columbia University, New York, New York, USA

**Key Points:**

- Individuals with diabetes are at increased risk for COVID-19 and its complications.
- There are distinct phases to COVID-19.
- There are various testing approaches for COVID-19 with different benefits.

**David C. Kaslow, MD**

PATH, Seattle, Washington, USA

**Key Points:**

- SARS-CoV-2 vaccine development has been unprecedented in its magnitude and breadth of candidates, creating a high likelihood of success as well as challenges in downselection and decision-making for global public sector funding.
- The vaccine development effort has identified streamlined pathways for early development; however, late-stage development, licensure, policy, and financing approval pathways have yet to be tested.
- Challenges and opportunities in developing and deploying SARS-CoV-2 vaccines include the theoretical potential for vaccine-enhanced diseases, correlates of protection and risk, targeting at-risk populations (e.g., elderly, underlying disease, pregnancy), and allocation of vaccines.

**Nevan Krogan, PhD**

University of California, San Francisco, San Francisco, California, USA

**Key Points:**

- The SARS-CoV-2 human protein-protein interaction map reveals novel drug targets.
- Global phosphorylation analysis in infected cells identifies potential therapies targeting kinases.
- Common coronaviral host targeting mechanisms point to pan-viral therapies.

#### Summary of Panel

Individuals with diabetes and COVID-19 infection have an increased risk of worse outcomes and complications, as well as a two- to three-times increased risk of mortality. Possible mechanisms for worse prognosis include cytokine release through immune dysfunction and direct damage to beta cells, precipitating diabetic ketoacidosis (DKA).^2^ The clinical phases of infection are (1) pre-symptomatic, (2) viral symptoms, (3) cytokine storm, (4) coagulation, and (5) late hyper-inflammation, which can result in quadriplegia and vasculitis.^32^ Recent evidence has indicated that COVID-19 infection has a lingering effect, with 35% of individuals not returning to their usual state of health when interviewed two to three weeks after testing.^33^

CP has been used to treat COVID-19 by passively transferring antibodies from a convalescent individual into a recipient who is at risk of infection or already infected. However, use of CP is only a temporizing measure pending availability of other strategies for treatment and prevention,^34^ and results for treatment in COVID-19 infection are mixed. Observational studies generally show that treatment with CP is safe, well-tolerated, and associated with improvement in clinical status (weaning off of ventilation, improved oxygenation, reduced viral loads) and decreased mortality overall, especially with early use.^35-39^ Further studies are needed to evaluate efficacy.

**Figure 7.**
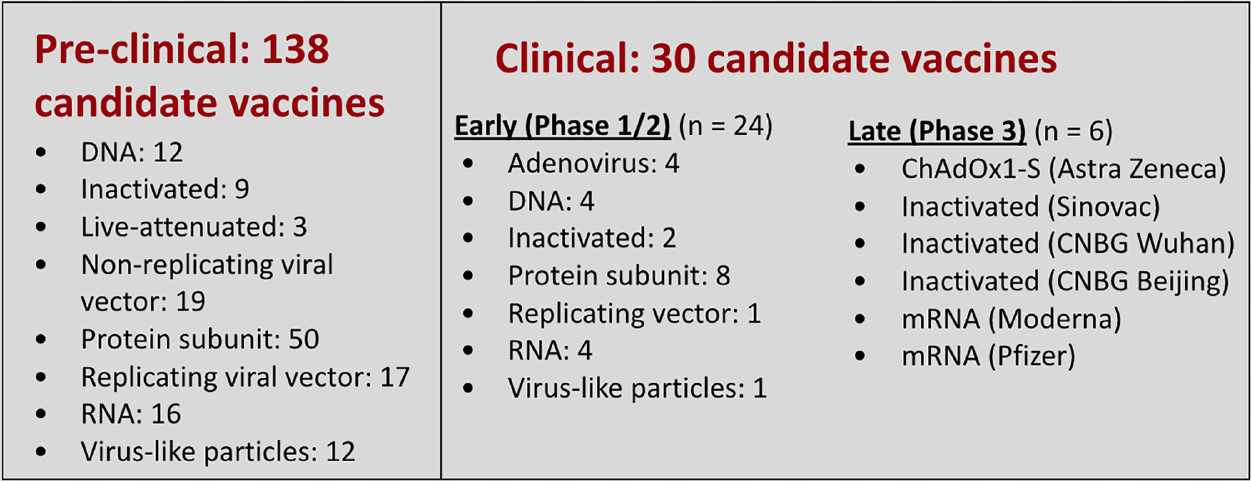
A landscape of SARS-CoV-2 vaccine development according to WHO, as of August 20, 2020. Figure provided by David C. Kaslow, MD, PATH Essential Medicines. Adapted from WHO landscape of SARS-CoV-2 candidate vaccines.^40^

SARS-CoV-2 vaccine development has been unprecedented in speed, breadth, and magnitude. There are at least 138 vaccine candidates now in pre-clinical evaluation. Two dozen vaccines are already in early development and six are in phase three trials.^40^ A landscape of SARS-CoV-2 vaccine development according to WHO, as of August 20, 2020 can be found in Figure 7. However, significant challenges must be overcome to develop and deploy a vaccine,^41^ including: (1) decision-making on global public sector funding, (2) accounting for the theoretical potential for vaccine-enhanced diseases, (3) establishing correlates of protection and risk, (4) targeting at-risk populations, and (5) determining allocation of vaccines.

The Quantitative Biosciences Research Institute (QBI) at University of California, San Francisco, is working to identify drug targets for COVID-19. The QBI Coronavirus Research Group (QCRG) was involved in generating a SARS-CoV-2 human protein-protein interaction map. Over 330 human proteins have been identified that are necessary for viral infection. Currently, 69 drugs and compounds have been identified that target these proteins, and a number of these have potential for being potent antivirals.^42^ Similarly, global phosphorylation analysis in infected cells can identify potential therapies using kinase inhibitors.^43^ Scientists have pinpointed several drugs that block the virus in lab tests, some of which are now in clinical trials. One drug, that looks particularly promising and was recently approved by the Food and Drug Administration (FDA) for clinical trials, is Zotatifin,^44,45^ a translational initiation inhibitor. In the future, more drugs will need to be transitioned into clinical trials, and the interactions between different drugs should be studied. The role genetics plays in SARS-CoV-2 infection and treatment should also be examined.

#### Section 6: Psychology of Patients and Caregivers

**Korey Hood, PhD**

Stanford University, Stanford, California, USA

**Key Points:**

- The COVID-19 pandemic has caused an increase in anxiety and distress in the general public.

- People with diabetes may have trouble accessing the resources they need because of the pandemic, worsening their anxiety and distress.
- People with diabetes should be encouraged to employ simple behavioral strategies to combat feelings of distress.

**William Polonsky, PhD, CDCES**

Behavioral Diabetes Institute, San Diego, California, USA

**Key Points:**

- People with diabetes are at elevated risk for distress and depression.
- People with diabetes are appropriately worried about COVID-19, which likely exacerbates distress and depression.
- Healthcare professionals (HCPs) can help by labeling and normalizing their patients’ concerns and encouraging a compassionate conversation about risk.

#### Summary of Panel

People with diabetes have an increased risk at baseline for depression, anxiety, and other psychological issues that can make it difficult to care for themselves. Diabetes in itself can be a psychologically taxing disease, and when you layer on it all the necessary alterations in lifestyle and accessibility to services that come with the pandemic, it is understandable that patients with diabetes will be affected. There are not many published reports so far on the magnitude of the psychological impact of the COVID-19 pandemic on people with diabetes. However, there are many reports^46,47^ showing increased anxiety, depression, and distress in the general population of adults and children. Likely predictors for increased psychological stress in this population include a change in social support (less, different types), limited resilience, being already overwhelmed (life stress, poverty, and baseline depression), increased emphasis on achieving better glycemic control, and trouble accessing diabetes care teams. HCPs can help in a number of ways. First, HCPs should label and normalize their patients’ concerns. Patients should be assured that it is understandable and reasonable that they feel this way. Next, providers should encourage a compassionate discussion about risk and help patients identify needs and safety precautions. HCPs can help by recommending simple behavioral strategies to their patients, which may include encouraging patients to follow their daily routines as much as possible, to reach out for social support as needed, and to engage in physical activity.

## Session III: Response

### Part C: Outpatient Care

Moderator: David T. Ahn, MD

Hoag Hospital, Newport Beach, California, USA

#### Section 7: Outpatient Treatment of Diabetes Mellitus and Non-Pharmacologic Intervention

**Nicholas Argento, MD, FACE**

Maryland Endocrine, Columbia, Maryland, USA

**Key Points:**

- Patient and staff preparation for effective remote diabetes visits: comprehensive data gathering by the patient with coaching by staff before the visit will improve the quality of the visit.
- Assessing glycemia is “all about the numbers,” so remote connection and HCP access to meaningful glycemic data, facilitated by cloud-based systems, are critical to allow optimal interventions.
- Seeing patients in the office requires careful assessment of local conditions where the patient lives, how they must travel, the location of the diabetes facility, and setting up a clinic environment that protects patients and staff. This can be done by emphasizing pre-visit risk screening, physical distancing, effective masking, avoiding patient grouping, enhanced cleaning of surfaces, and minimizing administrative tasks that can be carried out remotely.

**Frank Best, MD**

Die Diabetes-Praxis, Essen, Germany

**Key Points:**

- For diabetologists, it is important to train patients on using video conferencing tools, getting data out of their devices to share with their diabetes team, and bringing proper materials for hospital stays. It is also important to similarly train the diabetes team.
- For politicians, it is paramount to stop cutting expenditures for healthcare and to start investing in public health. A sufficient information technology (IT) infrastructure and proper personal protective equipment (PPE) must be provided for clinics and hospitals.
- Current lifestyles and globalization might foster the next pandemic.

**Anders Carlson, MD**

International Diabetes Center, Minneapolis, Minnesota, USA

**Key Points:**

- The COVID-19 pandemic has required clinical research to rapidly adapt.
- Diabetes and related COVID-19 risk factors will likely be an intense area of research in the coming years.
- More telehealth/virtual care may lead to broader participation in clinical research.

**Curtiss Cook, MD**

Mayo Clinic Arizona, Scottsdale, Arizona, USA

**Key Points:**

- There is possible overlap between T2D treatment and SARS-CoV-2 pathways.
- There is no evidence that T2D outpatient treatment regimens place the patient at greater risk of infection or worse outcomes of infection.
- There are no recommendations to change the T2D outpatient treatment regimen when a patient tests positive for SARS-CoV-2.

**Deborah Wake, MBChB, BSc, PhD, Clin Ed Dip**

University of Edinburgh, National Health Service Lothian, Scotland, UK

**Key Points:**

- Clear coordinated national/organizational policies on outpatient management during COVID-19 are essential to standardize care approaches and to take account of best practice. These should evolve over time and be well-communicated.
- Patient triage and risk stratification are essential to prioritize care delivery and limit requirements for face-to-face clinical contact.
- Simple technologies and digital solutions should be embraced to support “at-home” complications screening, remote monitoring, and patient education.

#### Summary of Panel

The traditional outpatient diabetes interaction has been completely disrupted by the COVID-19 pandemic, but its fundamental principles remain unchanged. Table 3 lists six actions for conducting clinical research to protect subjects who are participating in clinical trials during the COVID-19 pandemic. Reducing the complications of diabetes through self-management education and medications has always been the overarching goal of diabetology. Because poorly controlled diabetes is associated with an increased risk of adverse outcomes in patients with COVID-19, the pandemic provides a heightened sense of urgency for our patients to optimally manage their blood sugars and to improve other contributing comorbid conditions, such as obesity.

**Table 3.** Six Actions for Conducting Clinical Research to Protect Subjects Who Are Participating in Clinical Trials During the COVID-19 Pandemic.

Identify key members of the Institutional Review Board to quickly accommodate protocol changes and approval for essential visits
Use video or phone as much as possible, having participants come in only for essential study activities (such as electrocardiography, phlebotomy, etc.)
Work remotely with participants to download devices from home as much as possible
Implement Health Insurance Portability and Accountability Act-authorized electronic-signature methods to avoid print-and-sign
Convert many paper forms to “fillable” forms that could be completed remotely
Arrange with the IT department to allow study monitors remote access to the electronic medical record

Table provided by Anders Carlson, MD, International Diabetes Center. Abbreviation: IT, information technology

Drastic measures to reduce the transmission of COVID-19, such as stay-at home orders, face masks, and social distancing, have forced clinicians to rethink the way diabetes care is delivered. The initial response has come largely in the form of virtual telemedicine visits, enabled by the data-sharing capability of digital diabetes tools such as continuous glucose monitors (CGMs) and insulin pumps. Furthermore, triage algorithms have been introduced to assist with prioritizing which types of patients should be evaluated in a timely manner and which can be postponed.^48,49^ Such algorithms might potentially be configured to empower the management of entire populations by better allocating limited resources to patients most in need. Finally, the reach of remote care is growing to include patient education, clinical research, and even some routine screening services such as specimen collection.

#### Section 8: Technology and Telehealth for Diabetes Outpatients

**Eirik Årsand, PhD**

UiT The Arctic University of Norway, Tromsø, Norway

**Key Points:**

**Figure 8.**
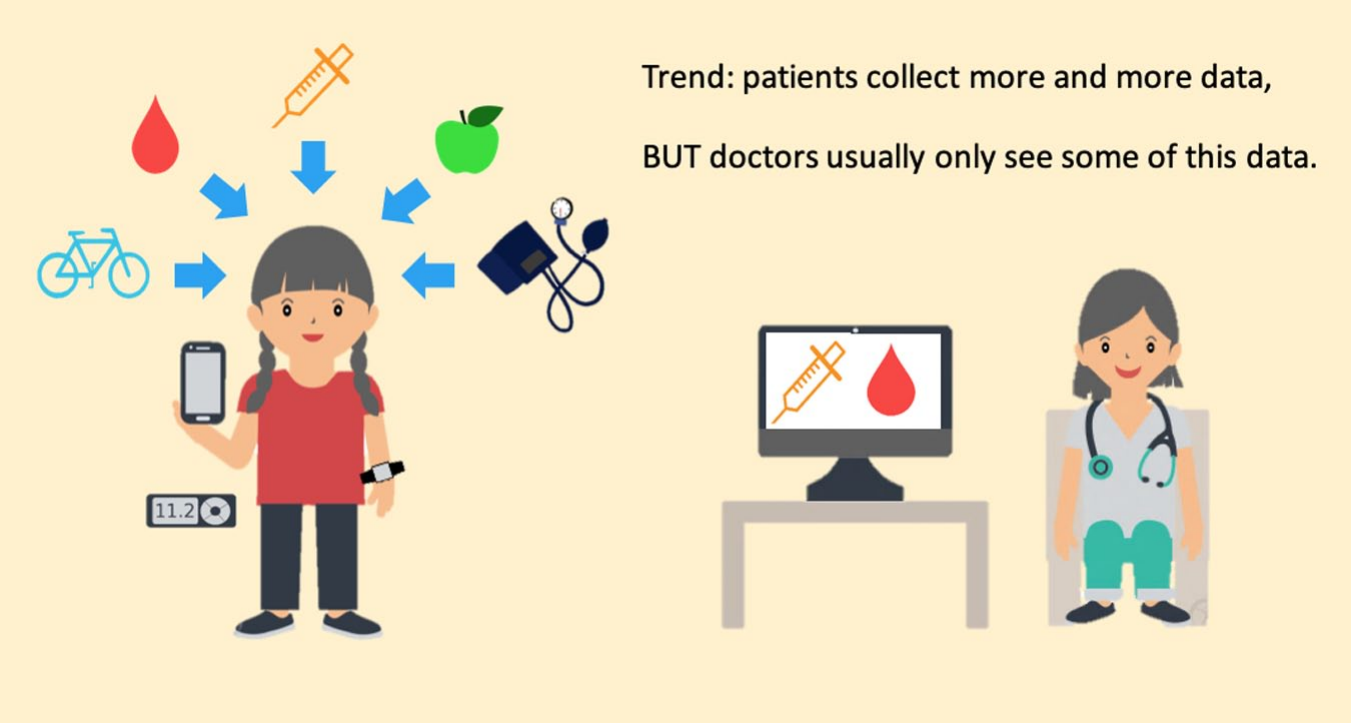
The increased amounts of data that patients track using various smart devices compared to the patient data that doctors have access to. Figure provided by Eirik Årsand, PhD, The UiT Arctic University of Norway. Adapted from Bradway/Årsand, Norway 2020.

- Health personnel need time, information, and training on how to use social media to understand the patients’ use of self-management tools—including those tools patients make themselves and use outside the standard offers from the healthcare industry.
- The way diabetes consultations are performed needs to change, not only during pandemics like COVID-19, but as a standard. Remote consultations need to be an option for all users and all consultations, and the concept of a consultation should include a “before,” a “during,” and an “after” part.
- More and more patients are now collecting relevant health information in addition to data on blood glucose (BG) and medication use as part of their daily lives (Figure 8). This additional patient-gathered data should be used when patients and HCPs meet.

**Juan Espinoza, MD, FAAP**

Children’s Hospital Los Angeles, University of Southern California, Los Angeles, California, USA

**Key Points:**

- Beyond encounter-based telehealth: billing and reimbursement options for more continuous care.
- Meeting patients where they are: language, technical, and access concerns for technology-enhanced care.
- Maturity models: are providers ready for technology-enhanced care?

**Aaron Neinstein, MD**

University of California, San Francisco, San Francisco, California, USA

**Key Points:**

- The COVID-19 pandemic has expedited a preexisting trend toward increased use of telehealth for diabetes care delivery, leveraging changes in delivery system infrastructure and workflow, federal reimbursement policy, and patient and HCP expectations.
- Next steps needed to improve video visit quality and experience are better workflows and technologies to ensure pre-visit device data connectivity and availability, in-visit screen sharing and annotation, and electronic health record-integrated diabetes device data to facilitate efficient HCP review, documentation, and reimbursement.
- Ultimately, care models, technologies, and workflows are needed to support continuous diabetes care, including personalized follow-up check-ins between visits and population-based patient identification and outreach.

**Kirsten Nørgaard, MD, DMSc**

Steno Diabetes Center, Copenhagen, Denmark

**Key Points:**

- My hospital, Steno Diabetes Center Copenhagen
  ○ A large public outpatient diabetes clinic (9500 diabetes patients)
  ○ Technology mainly used in T1D
  ○ Telemedicine consultations increased after COVID-19
- Training patients in device upload and data review is as important as training them in using the technology. Thus, include such training when starting technology!
- In Europe, a noncommercial, shared uploading platform for all device brands is not available for clinics.

#### Summary of Panel

The widespread, rapid implementation of telehealth has been a bright spot amidst the darkness of the COVID-19 pandemic. With the increased amount of data that patients track using various smart devices (Figure 8), there is the potential of remotely accessing more types of patient data by HCPs, even after the pandemic. Diabetes is particularly well-suited for remote and asynchronous care thanks to the growing use of smartphone-connected tools such as CGMs, insulin pumps, and smart insulin pens. Furthermore, reimbursement codes now exist in the USA that incentivize providers to provide services such as virtual check-ins (G2012, G2010) and chronic care management (99490, 99487, 99489, G0506).

However, innovation alone is not the answer. Any solution must be designed from idea to execution with a clear focus in mind. For example, while technology is often looked to as an equalizer for social or economic disparities in healthcare, these barriers can only be overcome with intentionality. Otherwise, technology can, in fact, further widen the gaps between those with resources and those with limited access. Another pain point with diabetes technology originates from diabetes device companies that force patients to keep their health data within their own ecosystems, creating dreaded “data silos.” This practice is hostile to patients and can only detract from their care by making it harder to integrate multiple streams of data that are necessary for clinical decision-making.

Healthcare systems should seize the opportunity and be able to emerge from this pandemic better prepared for future unexpected challenges with innovative care delivery models, combining the best qualities of in-person and virtual visits, real-time and asynchronous care, and digital and analog solutions.

### Part D: Inpatient Care

Moderator: Amisha Wallia, MD, MS

Northwestern University Feinberg School of Medicine, Chicago, Illinois, USA

#### Section 9: Technology for Inpatients

**Elias Spanakis, MD**

University of Maryland School of Medicine, Baltimore, Maryland, USA

**Key Points:**

- After consultation with an inpatient diabetes-endocrinology team (and assuming nursing staff is trained and comfortable using these systems), we could then offer patients with COVID-19 and diabetes the option to either initiate using CGM devices or continue using existing outpatient CGM systems.
- Remote-wireless CGM monitoring, like glucose telemetry or similar systems, has the potential to reduce point-of-care (POC) glucose testing, nursing staff exposure/risk for COVID-19 transmission, and PPE utilization.
- Continuous subcutaneous insulin infusion (CSII) and automated insulin delivery (AID) systems could potentially be used in selected individuals who do not have any contraindications, because these systems could possibly decrease nursing staff exposure and PPE use. They may also reduce workload by eliminating the need for insulin administration by the nursing staff.

**Kathleen Dungan, MD, MPH**

Ohio State University, Columbus, Ohio, USA

**Key Points:**

- Expanded glucose monitoring in hospitalized patients should consider important pre-analytical, analytical, and post-analytical sources of error.
- A hybrid approach to measuring glucose with a CGM and POC glucose monitor in the ICU can be considered in conjunction with risk mitigation measures.
- Implementation of using CGMs in the ICU in the COVID-19 era requires a collaborative/team-based, iterative approach.

**Joshua Miller, MD, MPH**

Renaissance School of Medicine at Stony Brook University, Stony Brook, New York, USA

**Key Points:**

- CGMs can help improve care for and monitoring of critically ill patients with COVID-19.
- CGMs can potentially help decrease PPE utilization and increase clinician safety during the COVID-19 pandemic.
- Glucometrics data provide valuable information about clinical outcomes in patients with hyperglycemia/diabetes and COVID-19.

#### Summary of Panel

**Figure 9.**
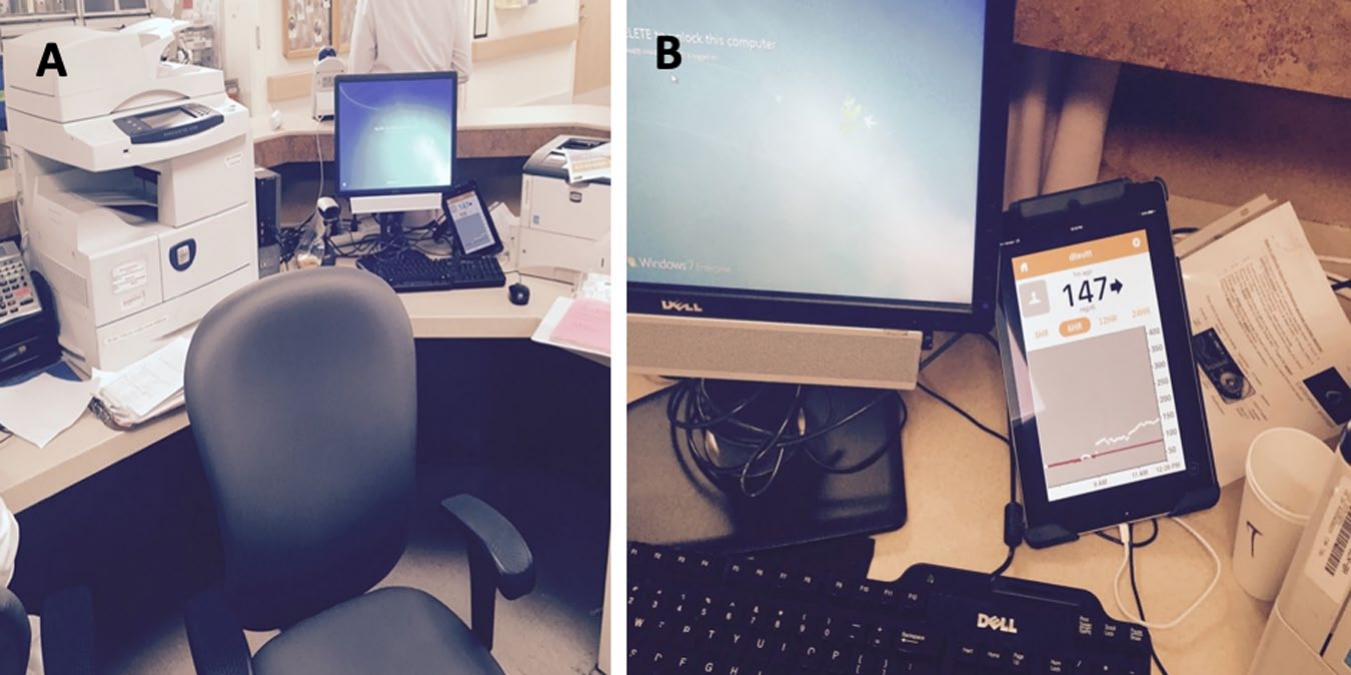
(a) An image and (b) a magnified image of a glucose telemetry system. Images were taken at the Baltimore VA Medical Center and provided by Elias K. Spanakis, MD, University of Maryland School of Medicine.

The COVID-19 pandemic has allowed for significant technological advancement in the inpatient care setting in a short period of time. The use of CGMs and remote wireless monitoring through glucose-telemetry has allowed for the potential for improved glycemic control,^50^ decrease in nursing workload, and decreased exposure risk.^51,52^ In April 2020, the FDA allowed discretion of enforcement for two CGM manufacturers to provide devices and technical support to hospitals and other healthcare facilities for off-label use to support COVID-19 healthcare-related efforts during the current pandemic.^53,54^ It is unclear how long this enforcement discretion will last. An example of a glucose telemetry system is shown in Figure 9. Patients with COVID-19 infection and diabetes could initiate using CGM devices or continue using existing outpatient CGM systems after consultation with appropriate inpatient teams (diabetes service/endocrinology) and once appropriate implementation (nurse training) has taken place.^54^ Several case reports/series have been published, demonstrating the initial feasibility of remote glucose monitoring and insulin adjustment based on monitoring with a CGM.^55-57^ In addition, CSII/AID systems could also be utilized in selected patient populations in both the ICU and floor settings to potentially improve glycemic outcomes and reduce work burden.^51,58^ However, implementation of any of these technologies requires an approach that is collaborative/team-based and allows for close follow-up and adaptation if needed over time. Known and unknown potential sources of error (environment, technique, interference, clinical states such as anemia and hypotension, or delayed results) need to be closely monitored.^59^ Implementation barriers, such as establishing appropriate algorithms, stakeholder alignment, technology components (Wi-Fi), and data integration, should be addressed prior to implementation. In the ICU, various approaches that use POC devices and CGMs have been evaluated. In the first phase, until sensor validation is obtained or until the first 24 hours, frequent POC glucose testing is performed, which is used for insulin adjustment. In the next phases, while the patient is still in the ICU, CGM readings are used for insulin titration, where POC testing is performed as adjunctive glucose measurements. In the final phase, when patients are transferred to the non-ICU setting, CGM readings are used mainly for insulin adjustment with POC performed infrequently, as needed. Glucometrics and use of glucose telemetry present an invaluable opportunity to monitor critical information about clinical outcomes in patients with hyperglycemia, diabetes, and COVID-19.

#### Section 10: Management for Diabetes Inpatients with COVID-19

**Shivani Agarwal, MD, MPH**

Montefiore Medical Center, Albert Einstein College of Medicine, Bronx, New York, USA

**Key Points:**

- Incorporating data related to treatment of hyperglycemia/DM in COVID-19 into real-time clinical decision-making can lead to improved outcomes.
- Adaptation and implementation of diabetes care protocols in COVID-19 is a priority.
- Opportunities to use technology for inpatient diabetes management should be leveraged.

**Joseph Aloi, MD**

Wake Forest School of Medicine, Winston-Salem, North Carolina, USA

**Key Points:**

- Inpatient glycemic management consults for COVID-19 patients are common.
- Remote glycemic management is practical and expands access to specialized care.
- Access to technology facilitates transitions of care from ICU-to-floor and floor-to-home.

**Francisco Pasquel, MD, MPH**

Emory University, Atlanta, Georgia, USA

**Key Points:**

- Uncontrolled diabetes in the hospital is associated with severity of disease and mortality in patients with COVID-19.
- To preserve PPE, reduce exposure, and improve care, changes in inpatient diabetes protocols are being developed globally.
- The use of diabetes technology in the hospital is rapidly evolving for non-ICU and ICU patients and may help decrease the burden of diabetes care during this pandemic.

**Robert Rushakoff, MD**

University of California, San Francisco, San Francisco, California, USA

**Key Points:**

- Do not make wholesale changes to your insulin protocols. The more changes you make, the higher the risk for potential errors.
- Insulin requirements can be very high, but generally intravenous (IV) insulin infusions are not needed, and IV lines frequently clot off. Instead, rapid acting insulin given every four hours may be safely used; this dose can be very high in these patients, sometimes greater than 30 units every four hours to maintain glucose in range.
- Cluster care can be used in both the ICU and acute care units. Generally, glucose checks and insulin dosing can be clustered with other scheduled nursing interventions, thus reducing exposure and potentially improving care.

#### Summary of Panel

There has been a global response to preserve PPE, reduce exposure, and improve care by adapting diabetes/hyperglycemia inpatient protocols. Protocol sharing along with local, national, and international efforts of describing and collating cases are also underway.^60,61^ In addition, technology can be leveraged to obtain data safely and then utilized for real-time clinical decision-making with the goal of improved outcomes. Applying data for risk stratification could streamline care; as an example, previous insulin use has been strongly associated with COVID-19 mortality, while being older, male, and obese has also increased mortality risk for patients with COVID-19.^62^ Protocols, which could specify use of subcutaneous insulin for treating DKA, can be utilized for COVID-19-related care with the goal of both preserving ICU beds and decreasing face-to-face time.^60,62^ However, both utility and implementation successes and failures across systems and across countries will need to be studied closely.

Inpatient glucose/diabetes consultations are clearly increasing in the COVID-19 era, and remote glycemic management can expand access, while allowing for both practicality and speed without forsaking quality. Virtual care is also being explored, and telehealth and virtual visits could deliver optimal care regardless of circumstance, while also allowing for increased capacity. Bedside tablets, e-consults, virtual glucose management services, and even phone calls can be utilized to complete telehealth visits or hybrid visits day-to-day depending on the hospital volume.

While technology can be critical to improving care during these challenging times, the potential benefit of any new interventions or workflows must be weighed against the possibility of introducing new safety risks. Major changes that do not rely on known infrastructure and training could increase the risk of errors. Simple approaches such as clustering care in both ICU and other care units should be used, and special efforts should be made to cluster glucose checks, food delivery, insulin dosing, and delivery of other potentially necessary interventions.

#### Section 11: Ethics

**Jacob M. Appel, MD, JD, MPH**

Icahn School of Medicine at Mount Sinai, New York City, New York, USA

**Key Points:**

- Serious consideration should be given to altering standards regarding withdrawal of care during a pandemic to ensure that resources are allocated in a rational and life-preserving manner.
- Crisis standards should be uniform among states in the USA to facilitate the transfer of scarce resources such as ventilators during a pandemic and to ensure equity between jurisdictions.
- Researchers should tolerate higher risk studies (including human challenge trials) during a pandemic when a “trolley problem” scenario exists and altruistic volunteers are willing to accept higher levels of danger for vastly enhanced potential societal benefit.

**James Tabery, PhD**

University of Utah, Salt Lake City, Utah, USA

**Key Points:**

- Crisis standards of care (CSC) triage protocols evolved nationwide throughout the spring and summer of 2020 in response to concerns raised about age and disability discrimination.
- Despite the updates to the CSC triage protocols, ethical and legal worries remain.
- Some CSC triage protocols continue to pose the risk of having a disparate impact on certain patients with certain health conditions, including diabetes.

#### Summary of Panel

The COVID-19 pandemic and the need for resources, including ventilators, hospital beds, and even trained medical professionals, has highlighted the necessity of understanding and ethically guiding care during this pandemic. The pandemic puts policymakers in a “trolley problem” scenario, a famous ethical scenario in which there exists a choice to sacrifice a few to save many. If there are volunteers, then higher risk studies should be pursued to maximize societal benefit. Healthcare has invisible and visible victims, and in crisis times such as the COVID-19 pandemic, these victims and their needs may converge. Altering standards can be one approach to ensure that resources are allotted in a ubiquitous and life-preserving manner. It is critical that health inequities are not widened, but also that new health inequities are not created. One notable example is making crisis standards uniform among states, so that the transfer of resources (ventilators, personnel) can occur to allow for proper allocation of resources, making locations potentially more equitable.

The COVID-19 pandemic has left some areas in the USA with scarce resources. In response, CSC triage protocols from the past were reinstituted with the goal to provide more utilitarian care. These CSC triage protocols evolved throughout the USA to lessen discrimination, but concerns were still raised about age and disability discrimination, including against chronic diseases such as diabetes. Initial assessments have now replaced specific criteria for triage to address some of these concerns. It is possible this could have a greater and potentially more disparate impact on our older patients or patients with diabetes, especially those older adults with diabetes.^63^ The need for equitable care must be balanced and should not allow for possible discrimination.

## Session IV: Recovery

### Part E: Resources

Moderator: Elias K. Spanakis, MD

University of Maryland School of Medicine, Baltimore, Maryland, USA

#### Section 12: Accuracy of Diagnostic Tests

**Jonathan Schmitz, MD, PhD**

Vanderbilt University, Nashville, Tennessee, USA

**Key Points:**

- COVID-19 highlights critical distinctions between tests for diagnosis, tests for screening, and tests that help identify a cure.
- The pandemic illustrates how, for some scenarios, rapid diagnostic expansion is key, while for others, diagnostic stewardship is critical.
- Beyond SARS-CoV-2, the pandemic has refocused attention on the regulatory framework by which diagnostic tests are developed in the USA.

**Nam Tran, PhD, HCLD (ABB), FAACC**

University of California, Davis, Sacramento, California, USA

**Key Points:**

- Reagents and consumables related to molecular SARS-CoV-2 testing remain severely limited in the USA. Mitigating the impact of supply chain challenges can include diversification of testing platforms, prioritization of testing, and adoption of novel testing schemes including pooling and POC testing.
- As the COVID-19 pandemic evolves, discrepancies between how analytically sensitive a test is versus the perceived clinical sensitivity have been observed. These discrepancies are influenced by specimen quality, viral kinetics, and specimen type.
- Diversification of COVID-19 diagnostic platforms is key. Specimen quality, viral kinetics, and specimen type influence clinical sensitivity of SARS-CoV-2 diagnostic assays. Serology testing should follow current CDC guidelines. Antigen testing shows promise; however, the lower sensitivity limits widespread applications.

#### Summary of Panel

**Figure 10.**
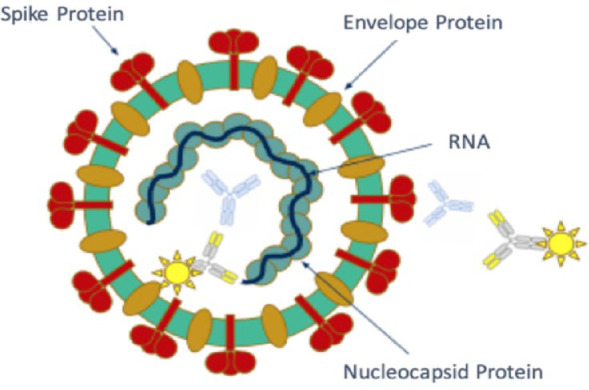
The viral structure of SARS-CoV-2 and three diagnostic targets is illustrated, including the (a) spike protein, (b) envelope protein, and (c) nucleocapsid protein. Blue antibodies targeting nucleocapsid and spike proteins are those produced by the patient, and yellow labeled antibodies are detection antibodies used for serology assays. Figure provided by Nam Tran, PhD, HCLD (ABB), FAACC, University of California, Davis.

It is difficult to define the accuracy of the different COVID-19 tests because direct comparisons of the various assays are not available. The viral structure of SARS-CoV-2 and three diagnostic targets are illustrated in Figure 10. While there are differences in the reported sensitivity rates (94.4% to >97.5%), reported specificity has been in an acceptable range. Some of the reasons that can explain the false negative results of the tests is the quality of the nasopharyngeal swab samples as well as the timing of the tests, especially in the early stages of the COVID-19 infection. This is because adequate viral load needs to be present for SARS-CoV-2 detection. Prolonged positivity poses another challenge because this makes testing harder to interpret in order to distinguish a cure from active disease. Overall, the performance of the nucleic acid amplification tests should be framed against their particular clinical context, depending on whether they are used for screening, diagnosis, or identifying a cure. Screening tests aims to detect infected individuals before symptoms develop, while diagnostic tests are used to confirm infection in individuals who are demonstrating symptoms.^64^ Serology assays represent additional options and can identify those who have been exposed (sensitivity 80%-97%). However, they are also limited because they only identify individuals who have produced antibodies. Antibody tests are highly specific for SARS-CoV-2 by detecting antibodies in a blood sample. The antibody target is usually either the nucleocapsid or spike proteins of SARS-CoV-2. Restrictive approaches to use serology testing for only selected individuals have been developed. Antigen assays are additional methods used for targeting viral proteins. Antigen tests, typically immunoassays, detect SARS-CoV-2 viral proteins in respiratory specimens. At this time, emergency use-authorized tests target symptomatic patients only (sensitivity 80%-87%), with negative results reconfirmed by molecular methods if necessary, as determined by a physician. Molecular tests detect SARS-CoV-2 viral ribonucleic acid (RNA) in respiratory specimens. Typically, molecular tests use the polymerase chain reaction (PCR) or a similar technology to amplify viral genetic material. In comparison to antigen tests, they have increased sensitivity. Rapid testing may have benefits, because these tests may detect SARS-CoV-2 infection earlier. POC testing enables faster detection and can help with isolation, PPE decisions, and contact tracing. Although rapid diagnostic expansion is key, diagnostic stewardship is also critical.

#### Section 13: Children

**Zoltan Antal, MD**

Weill Cornell Medicine, New York, New York, USA

**Key Points:**

- A significant proportion of hospitalized children with T1D and COVID-19 have been Black or Latinx.
- Hemoglobin A1c (HbA1c), insurance type, and CGM use are associated with hospitalization risk.
- DKA has been common among hospitalized COVID-19-positive children with T1D.

**Jennifer Raymond, MD, MCR**

Children’s Hospital Los Angeles, University of Southern California, Los Angeles, California, USA

**Key Points:**

- Telehealth can increase visit attendance, compliance with standards of care, and patient satisfaction, while improving psychosocial outcomes in people with diabetes in a cost-effective manner.
- The transition to telehealth during COVID-19 has highlighted the need for further creativity and intentional focus on addressing disparities in care.
- Advocacy for sustained changes in telehealth legislation and reimbursement is critical for continued excellent care of people with diabetes.

#### Summary of Panel

There is limited information about the effect of SARS-CoV-2 infection in children with diabetes, especially among those with T1D. Preliminary data from the T1D surveillance study have shed some light on this population.^65^ This study has aimed to gather clinical data from HCPs in different clinical sites across the USA for T1D patients who were suspected or confirmed to have COVID-19. The study includes questions related to clinical presentation, diabetes control mode (pumps or sensors), duration of diabetes, diabetes control (HbA1c), need for hospitalization, and complications (DKA, severe hypoglycemia, ICU admission, death). Children who tested positive for COVID-19 infection (*n* = 37) (compared to those who had COVID-19-like symptoms but negative PCR test results for COVID-19 infection [*n* = 38]) were more likely to have higher HbA1c concentrations, public insurance, and new onset of T1D. These children were also more likely to be hospitalized for DKA and less likely to use insulin pumps. Among those who were confirmed COVID-19 positive, 80% of those hospitalized were Black or Latinx. Those who were hospitalized (*n* = 20) were more likely to have public insurance, less likely to use CGMs, and had higher DKA rates.

Therefore, improving care among children with diabetes is extremely important during the current crisis. Evidence from the pre-COVID-19 era showed that telehealth can be a promising tool among adolescents and young adults with T1D, because it leads to increased visit frequency and improved psychosocial outcomes without increasing total cost. During the current COVID-19 period, those patients who were monitored by telehealth missed fewer appointments and had similar satisfaction to that of those being seen in person. Additionally, HCPs experienced higher satisfaction rates with telehealth compared with in-person care. However, in order to widely adopt telehealth, we need to overcome socioeconomic and technological barriers and challenges. We need to (1) design new models for providing patient support, (2) advocate for permanent legislative changes, (3) publish outcomes and treatment recommendations, and (4) focus on inequities and disparities in healthcare.

#### Section 14: Pregnancy

**James Bernasko, MD**

Renaissance School of Medicine at Stony Brook University, Stony Brook, New York, USA

**Key Points:**

- The COVID-19 pandemic has directly and indirectly caused significant disruption to medical access and pregnancy care protocols.
- Regional experiences differ slightly on the extent to which pregnancy per se worsens prognosis, but outcomes appear generally good.
- Medical care practitioners should be willing to think and function “outside the box” if pregnancy care and outcomes are not to be significantly compromised.
- All pregnant and/or lactating women should adhere as closely as possible to current protocols to prevent SARS-CoV-2 transmission.

**Lynn M. Yee, MD, MPH**

Northwestern University Feinberg School of Medicine, Chicago, Illinois, USA

**Key Points:**

- The antenatal period is a time of intensive health services utilization. The abrupt transition to telehealth raises new challenges for women with pre-gestational and gestational diabetes mellitus (GDM), including late diagnosis of GDM and missed postpartum diagnosis of T2D.
- Pregnant women who are at greatest risk of pre-gestational and GDM are also at greatest risk of COVID-19 acquisition, and the intersecting SDoH can amplify the risk of complications from both conditions.
- The digital divide is particularly challenging for pregnant women because many do not have access to technology-enabled BG monitors, consistent home internet access, or other tools that allow easy communication about glycemic control outside of in-person visits.

#### Summary of Panel

Data about COVID-19 and pregnancy are accumulating rapidly; however, there is limited information about pregnant women with COVID-19 infections who also have diabetes. Women who are pregnant and have COVID-19 infections report similar frequency of cough (>50%) and dyspnea (30%) and fewer occurrences of headaches, muscle aches, fever, chills, and diarrhea in comparison to non-pregnant women with COVID-19 infections.^66^ Pregnant COVID-19 patients have a higher risk of preterm and cesarean delivery. Higher BMI values were found to be associated with more severe disease and adverse outcomes.^67^ Trans-placental transmission of SARS-CoV-2 can occur,^68^ and SARS-CoV-2 has been also identified in human milk.^69^

The COVID-19 pandemic changed the way that we manage pregnant patients with diabetes. Telemedicine visits increased from 0% to 60% of all routine prenatal and postnatal care visits (James Bernasko: unpublished data). Maternal surveillance is performed by reviewing glucose logs remotely, while fetal surveillance has to be individualized, based on maternal glycemic control. Healthcare challenges have led to delayed diagnosis of GDM, delayed treatment, and decreased time to prevent complications. The current crisis has made lifestyle changes difficult to achieve, because food access has become a major concern. Limited food access is due to financial and other constraints. The pandemic has also made it harder to exercise at a gymnasium. Enhanced access burdens have been described, including (1) delayed entry to prenatal care or enrollment in pregnancy-based Medicaid, (2) reduced availability of social support services, and (3) greater challenges accessing medications. Telehealth disparities pose important challenges because many low-income pregnant patients with diabetes cannot afford wireless Bluetooth-enabled BG monitors and have limited or no in-house internet connection, leading to decreased healthcare access.

#### Section 15: Economics of Care for COVID-19

**Lynn Barr, MPH**

Caravan Health, Kansas City, Missouri, USA

**Key Points:**

- There was a move to virtual care because patients stopped coming in for visits during the pandemic. The economics of this shift did not work out for HCPs.
- There has been a loss of primary and preventative care services associated with healthcare avoidance and reduced access that have been precipitated by layoffs.
- Significant regional variability in healthcare performance exists, so value-based payment calculations should ideally not be reliant on regional healthcare data from 2020.

**Paul Gerrard, MD**

McDermott+Consulting, Washington, DC

**Key Points:**

- Achievement of effective diabetes management requires that assessment and treatment both prevent complications and recognize complications when they are present. Prevention relies on high frequency/low resource interactions (eg, frequent blood sugar monitoring, blood pressure monitoring). Addressing complications requires the addition of low frequency/highly resource intensive services (eg, vascular surgery, hospitalization for DKA, dialysis).
- Historically, reimbursement paradigms in medicine have been developed around low frequency resource intensive services. These have been better suited to address the complications of diabetes than to address the underlying disease prior to the development of complications.
- Shifting reimbursement paradigms toward high frequency/low intensity, including the formalization of remote physiologic monitoring and, under the public health emergency, availability of expanded access to telehealth services, may permit reimbursement paradigms that align better with the management of diabetes.

**Wei-An (Andy) Lee, DO**

Los Angeles County+USC Medical Center, Los Angeles, California, USA

**Key Points:**

- The emergent implementation of telehealth in the Medicaid population in Los Angeles County has revealed the benefits of telehealth, including benefits for patients who require regular follow-up from their physicians. However, there are also many barriers to telehealth, including a lack of good outcome data and adequate internet access.
- Not investing in the last mile to bridge the digital divide will bar patients from receiving digital health services.
- Key investments are needed for the implementation of telehealth for successful adoption of telehealth in the post-pandemic era.

**Ateev Mehrotra, MD, MPH**

Harvard University, Boston, Massachusetts, USA

**Key Points:**

- Telehealth use rose rapidly early in the pandemic and since then has plateaued.
- Surprisingly, despite concerns about the digital divide, patients in poorer communities, compared to those in urban communities, were not less likely to use telehealth.
- Cognitive specialties, such as endocrinology, compared to procedural-based specialties, have embraced telehealth much more enthusiastically.

#### Summary of Panel:

**Figure 11.**
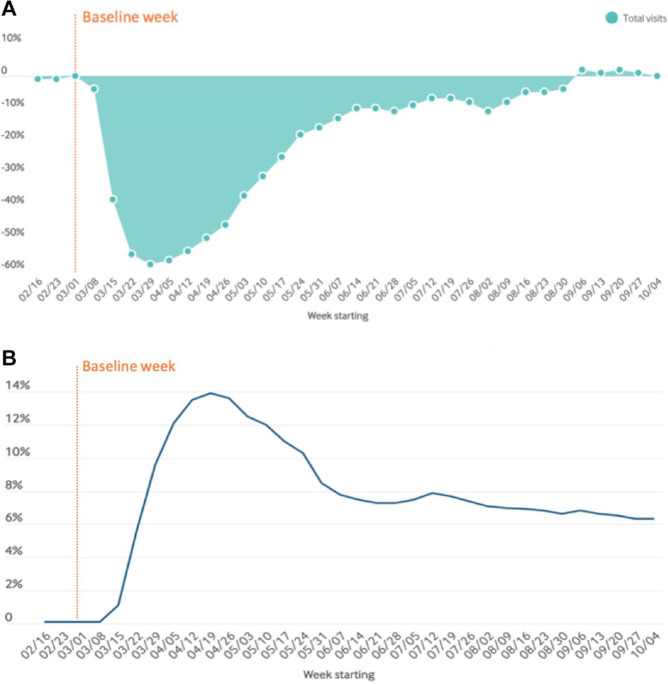
The total number of outpatient visits and the percentage of these visits that were by telemedicine since the beginning of the pandemic. (a) A graph of the percent change in the number of outpatient visits (compared to baseline) from March 1, 2020 through October 4, 2020. Dates are on the x axis, and the percent increase or decrease in the number of visits is on the y axis. (b) A graph showing the proportion (as a percentage) of total outpatient visits using telemedicine in the USA each week from March 1, 2020 through October 4, 2020. The axes in (a) and (b) have the same units. Figures adapted from “The Impact of the COVID-19 Pandemic on Outpatient Care: Visits Return to Prepandemic Levels, but Not for All Providers and Patients.”^70^

Managing diabetes-related complications requires collaboration of multiple specialists and utilization of high-resource intensive services, although relatively infrequently. A better approach is to utilize low intensity services with higher frequency, an option that telehealth can offer, with a goal of preventing rather than treating diabetes-related complications. During the COVID-19 crisis, total outpatient visits decreased overall, as illustrated in Figure 11(a). In contrast, because of implemented changes by policymakers, the proportion of telemedicine visits rose rapidly early in the pandemic, then plateaued, and later slightly decreased, compared to the highest achieved levels. This pattern is illustrated in Figure 11(b). These changes were not seen in all specialties, because some HCPs saw an overall increase and others a decrease in the total number of healthcare visits in their specialties. Key factors that led to telehealth success were (1) reduction of barriers to access care, (2) increased opportunities for patients to be more engaged with their healthcare, (3) frequent follow-up visits, (4) easier medication titrations, and (5) decreased no-show rates. Even for the subset of patients that require face-to-face visits, those individuals can be more easily identified and stratified at the initial telehealth visits. Two problems can limit adoption of telehealth: (1) some HCPs, as well as patients, may not have the skills to use telehealth technology, and they may have difficulties connecting with each other by phone or internet; (2) disadvantaged populations may have restricted or no digital access, limiting their ability to use telehealth. Five key barriers to delivery of telehealth in a healthcare system serving a Medicaid population are presented in Table 4.

**Table 4.** Five Key Barriers to Delivery of Telehealth in a Healthcare System Serving a Medicaid Population.

Strong reliance on the physical exam to diagnose and manage conditions that cannot be done through telehealth. Example: need for vaccinations, and/or procedures that cannot be done remotely.
Difficulty onboarding HCPs and patients.
Difficulty in navigating telehealth technology through legacy workflow of the healthcare system.
Difficulty when translation is needed during the telehealth exchange.
Difficulty reaching patients in their homes with phone or internet.

Table provided by Wei-An (Andy) Lee, DO, Los Angeles County+USC Medical Center. Abbreviation: HCPs: healthcare professionals.

### Part F: High-Risk Groups

Moderator: Lauren E. Wisk, PhD

University of California, Los Angeles, Los Angeles, California, USA

#### Section 16: Role of Industry

**Daniel Cherñavvsky, MD**

Senior Director of Medical Affairs, Dexcom, San Diego, California, USA

**Key Points:**

- The COVID-19 pandemic made clear the need for saving PPE and minimizing patient contact. Implementing use of CGMs in hospitals would help achieve these goals.
- More than 190 hospitals and health systems from all around the USA have inquired about the use of real-time (rt-)CGMs for inpatients. In addition, several educational activities such as webinars by ADA and Medscape have been used to educate interested parties on the benefits of using rt-CGMs.
- Moving forward, a national registry has been created to collect data on the performance of rt-CGMs used for hospitalized patients. In order to continue the use of rt-CGMs after the pandemic, it will be necessary to gather data for regulatory agencies.

**Corinne Fantz, PhD, DABCC, FACB**

Director of Medical and Scientific Affairs-POC, Roche Diagnostics, Indianapolis, Indiana, USA

**Key Points:**

- The diagnostic industry is critical to advancing the practice of medicine to improve the lives of people with diabetes.
- Driving diagnostic innovation and challenging the regulatory status quo is how we are moving the needle in the COVID-19 pandemic.
- Developing novel tests to diagnose, manage, and treat patients is what in vitro diagnostic industry partners are doing to address the COVID-19 global threat.

**Rosalind Hollingsworth, PhD**

Global Medical Franchise Head, Influenza, Global Medical Affairs, Sanofi Pasteur, Swiftwater, Pennsylvania, USA

**Key Points:**

- As the world is tackling the ongoing pandemic, vaccines are essential in the fight against SARS-CoV-2, to help protect the vulnerable and to allow communities to “return to normal.”
- The global need for vaccines is massive, so no single vaccine or company can meet the full demand. Unprecedented collaboration within industry and between industry and academia on vaccine candidates is needed.
  ○ As of August 2020, 168 candidate vaccines were being evaluated, according to WHO.^40^
- Expedited development pathways are being considered to rapidly bring to market effective vaccines without compromising safety. COVID-19 may peak in multiple waves and there is a risk that the virus will become seasonal.
  ○ Going into this and subsequent respiratory virus seasons, it is essential to maintain routine immunization practices for the protection of individuals and healthcare capacity (for example: influenza vaccines for those with diabetes).

**Jordan Messler, MD, SFHM, FACP**

Executive Director, Clinical Practice, Glytec Systems, Waltham, Massachusetts, USA

**Key Points:**

- Patients with COVID-19 who have stress hyperglycemia and/or diabetes have worse outcomes.
- IV insulin requires frequent BG checks and nursing intervention in the patient room. What solutions are helping frontline providers maintain best practices in the hospital?
- Technology-driven glycemic management can get patients into range faster and more safely and is proven to reduce rates of hypo- and hyperglycemia.

**Matthew Taylor, CFA**

MedTech Supplies & Devices Analyst, UBS-ARC, New York, New York, USA

**Key Points:**

- Given the vulnerability of people with poor glycemic control to COVID-19, the FDA exercised discretion to allow use of CGM systems for the treatment of patients in hospital settings and other facilities. Two CGM manufacturers initiated programs with several hospitals to make CGMs available to help monitor patients. Early data shows a trend toward reducing the incidence of low and high glucose events across all patients who used CGMs. Specific to COVID-19 patients, visits to patients’ rooms have been decreased by 30%-50% during their stays, saving equipment and reducing exposure for hospital staff.
- Improving the user experience, training, and ease-of-use with digital strategies: the diabetes technology players had already invested in digital strategies pre-COVID-19 to give patients access to information, allow for data sharing with caregivers/loved ones, and enable patients to upload data for use by HCPs. COVID-19 caused these players to accelerate these strategies to maintain continuity of care for patients using devices and to allow access for new patients in a virtual world. As a result of these strategies, diabetes device manufacturers (and patients) saw less disruption than many other areas of the healthcare sector through the early part of the pandemic.
- While access to testing remains below optimal levels, availability of tests should ramp up meaningfully through the fall, including greater access to rapid testing and tests that require smaller readers or no analyzers at all (lateral flow). A number of companies developed new-to-world assays that required complex clinical studies to get emergency use authorization (EUA) approval within just a few weeks or months. Typically, it is a three-year process to develop these assays, get them through clinical studies, and receive approval.

#### Summary of Panel

In this session, leaders in device manufacturing, vaccine development, diagnostic testing, and clinical management software discussed how their companies have rapidly shifted their focus during the COVID-19 pandemic to improving remote or distanced glycemic management in inpatient settings, preserving PPE, and driving diagnostic and therapeutic innovation. Dexcom worked with the FDA early in the pandemic to provide devices and technical support to hospitals who ordered CGMs for off-label use.^54^ They then provided training and support for hospitals using their system and tracked new COVID-19 cases to predict where hospital requests for CGMs would come from. Glytec Systems similarly expedited implementation of their insulin management software and rollout of software support. They are facilitating site collaboration for shared learning in the adoption of technology-driven management strategies. Roche Diagnostics is attempting to address the demand for diagnostic testing, which has continued to outpace supply, by striving for a faster turnaround of high-quality, consistent, and high-volume testing and bringing new innovative diagnostics to market. Sanofi has responded to the lack of an approved vaccine ready for sufficient distribution to meet global demand by facilitating collaboration between various companies and academia. Importantly, vaccine manufacturers noted that speed cannot compromise safety in vaccine development, but they are already preparing for eventual approvals. Finally, a market analysis revealed that the biggest technology shift during COVID-19 has come from CGM use. Expanded use of digital strategies for clinical management was noted to be a primary focus of future innovation in medical technology.

#### Section 17: Protection of Healthcare Workers

**Marissa Baker, PhD**

University of Washington, Seattle, Washington, USA

**Key Points:**

- Industrial hygienists use the hierarchy of controls when thinking about how to implement feasible and effective controls for a workplace exposure, including a viral exposure.
- The control methods at the top of the hierarchy are potentially more effective and protective than those at the bottom, which rely on worker compliance, but can be more challenging to implement.
- For a viral exposure, eliminating the virus through social isolation or vaccination is the most effective, but typically, the available controls are (1) engineering controls (eg, ventilation and physical barriers); (2) administrative controls (eg, work-from-home policies, staggering schedules, and training around COVID-19); and 3) PPE (eg, masks and face shields).

**Shuhan He, MD**

Massachusetts General Hospital, Harvard University, Boston, Massachusetts, USA

**Key Points:**

- In an emergency, digital and remote technology allow rapid organizational deployment.
- Just-in-time logistics, rather than just-in-case preparation, will be key going forward.
- Allocation algorithms are the newest way to ensure optimal resource deployment in limited settings.

**David Weissman, MD**

Centers for Disease Control, National Institute for Occupational Safety and Health, Morgantown, West Virginia, USA

**Key Points:**

- Protecting healthcare personnel from COVID-19 requires a comprehensive approach involving multiple types of interventions to limit exposures.
- CDC has published guidelines describing what to do when an HCP has had prolonged close contact with a patient, visitor, or other HCP with confirmed COVID-19, without wearing recommended PPE, and how they should thus be excluded from work for 14 days after their last exposure.
- Extensive up-to-date guidance on preventing transmission of COVID-19 in healthcare settings is available on the CDC website at https://www.cdc.gov/coronavirus/2019-nCoV/hcp/index.html.

#### Summary of Panel

**Figure 12.**
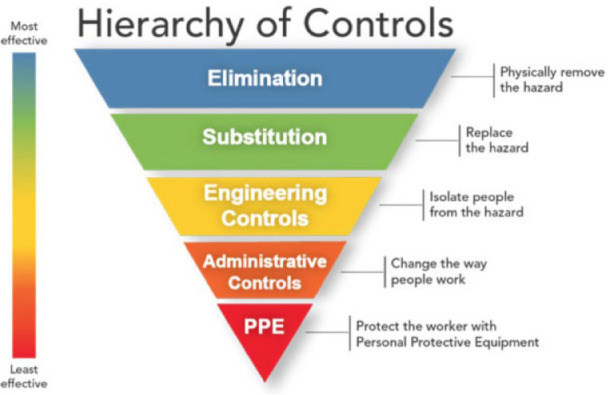
A hierarchy of five controls to mitigate or eliminate hazards, such as COVID-19, according to NIOSH. Control methods at the top of the figure are potentially more effective and protective than methods at the bottom.^74^ Abbreviations: COVID-19: coronavirus disease 2019; NIOSH: National Institute for Occupational Safety and Health.

Given the potential for occupational exposure to coronavirus among healthcare workers, panelists in this session began by describing the hierarchy of controls to protect against exposure in healthcare settings. The National Institute for Occupational Safety and Health (NIOSH) leads a national initiative called Prevention Through Design to prevent or reduce occupational injuries, illnesses, and fatalities through the inclusion of prevention considerations in all designs that impact workers.^71^ NIOSH recognizes a hierarchy of five controls to mitigate or eliminate hazards, which is depicted in Figure 12. From most effective to least effective, the five controls are as follows: (1) elimination, which removes a hazard (such as social isolation and vaccines), has been noted to have the greatest potential for effective control of contagious viruses; (2) effective substitution, which replaces a hazard with a less hazardous agent (such as by administering a drug that prevents viral replication and effectively substituting a less dangerous virus for a more dangerous virus) is currently not a viable control for COVID-19; (3) engineering controls, which isolate people from a hazard (such as ventilation and physical barriers), require time to implement; (4) administrative controls, which change how people act (such as telecommuting, staggered schedules, and hand hygiene), can be implemented quickly but depend upon compliance; and (5) PPE, which puts a barrier between a person and a hazard, serves as the least effective but most widely used control, and if the equipment fails, then the worker can be exposed to the hazard. Building on theoretical guidelines to control hazards, CDC has issued comprehensive guidance on optimizing PPE use.^72^ CDC also provides a variety of strategies that can be employed based on PPE capacity, from conventional (when supply meets demand) to contingency (anticipated shortages) to crisis (when supply cannot meet demand). Even with official guidance designed to plan for infection control and management, panelists noted that many healthcare organizations faced a sharp uptick in the need for PPE and a resultant shortage during the pandemic. Different facilities are known to have different needs and availability of PPE (for instance, well-resourced facilities or emergency rooms tend to have a greater supply, while smaller or hospice facilities tend to have a lesser supply). Early in the pandemic, organizations attempted to engage in manual exchange of equipment, but issues of speed and equity in manual allocation prompted the development of a nonprofit organization “Get Us PPE,” which provides donated PPE at no cost to frontline workers and under-resourced communities with a sense of urgency and a focus on equity.^73^ This organization employs matching algorithms to ensure optimal and efficient resource deployment. Organization data suggest early successes in efficient PPE allocation.

## Section 18: People with Diabetes

### Four Patients Who Contracted COVID-19

#### Summary of Panel

Four people with diabetes (two with T1D and two with T2D) discussed their experiences as individuals who contracted COVID-19. They described developing atypical symptoms (or what were considered atypical symptoms at the time they were diagnosed) during the disease course. They also reported, in some cases, to have severe prolonged symptoms after their recovery. While one person had a mild case of COVID-19 that was initially diagnosed as influenza, the other three reported a more severe illness, with two requiring hospitalization. Consistent with published clinical studies, their experiences emphasized that having good glycemic control was helpful for their recovery, but they also noted that the illness caused elevated glucose concentrations that were difficult to manage. Beyond the lasting physical effects of their infections, there was also a substantial impact on their mental well-being. Some experienced survivor’s guilt and anxiety/fear around the potential for reinfection. All four expressed gratitude for the care they received in the inpatient setting and from their regular HCPs during and after COVID-19.

## Session V: Surveillance

### Part G: Regulation

Moderator: Kong Chen, PhD, MSCI

National Institutes of Health, National Institute of Diabetes and Digestive and Kidney Diseases, Bethesda, Maryland, USA

## Section 19: International Responses to COVID-19

**Patricia Gomez, MD**

Universidad de Chile, Santiago, Chile

**Key Points:**

- South America is the new epicenter of the pandemic, with about five million confirmed cases and around 165,000 deaths, with Brazil being the most affected country to date, followed by Peru, Colombia, and Chile, which has high testing rates per million habitants.
- Isolation, quarantine, testing, and hospital planning measures were applied late. Poverty, social inequity, and difficulty in accessing health centers make the management of the pandemic more complex.
- During quarantine, a deterioration in glycemic control was observed. Newly implemented measures include special emphasis on primary healthcare, telehealth, distribution of medicines and supplies for three months or more, and medical appointments that follow all established disinfection and distancing protocols.

**David O’Neal, MD, FRACP**

St. Vincent’s Hospital, University of Melbourne, Victoria, Melbourne, Australia

**Key Points:**

- Our surveys indicated that, while initially (with lockdown) the patients attending our clinics with T1D preferred remote visits, after a few months, their preference was for face-to-face visits.
- Moving forward, however, the T1D patients expressed a preference for a mixed model of remote and face-to-face rather than one or the other.
- From an HCP perspective, the visits for those who were already engaged with insulin pumps and CGMs were more productive.

**Gerry Rayman, MD, FRCP**

Ipswich Hospital, East Suffolk and North East Essex National Health Service Trust, Getting It Right First Time Diabetes Clinical Lead, London, England

**Key Points:**

- Patients need speedy access to diabetes advice for (1) preventing hospital admissions, (2) receiving access to diabetes specialist care while being an inpatient, and (3) planning good supportive care after discharge to prevent readmission.
- This type of access requires integration of diabetes services and efficient organization.
- Existing guidelines needed to be adapted and presented in a brief and clear manner for use by non-specialists in the absence of specialists. Guidance from the UK Diabetes Inpatient COVID-19 Response Group is available at https://abcd.care/coronavirus.

**Eun-Jung Rhee, MD, PhD**

Kangbuk Samsung Hospital, Seoul, South Korea

**Key Points:**

- As of August 17, 2020, there were 16,058 COVID-19-positive patients and 14,006 patients have been negatively converted and released from quarantine. There have been 306 deaths since the outbreak of COVID-19. The case-fatality rate of COVID-19 patients in Korea is 1.9%. Most of the COVID-19 positive patients are asymptomatic.
- All asymptomatic COVID-19 patients are isolated in a government-managed facility. Once patients develop symptoms, they are transferred to a hospital with negative pressure ventilation wards. Our hospital has two negative pressure wards and two symptomatic COVID-19 patients are being treated there.
- I have COVID-19 screening clinic once a month, and usually see 10-15 patients with symptoms in one unit. The screening clinic is only for the patients who have symptoms or had contact with COVID-19 patients.
- From our analysis of the data released by the National Health Insurance System (NHIS), we discovered that diabetes patients have a 1.5- to 2.0-fold increased risk for death, oxygen therapy, and ICU care.
- COVID-19 patients in severe condition are being treated with supportive care with remdesivir and CP transfusion therapy.

**Laurien Sibomana, MS**

Rwandan Diabetes Association, Kigali, Rwanda, Africa

**Key Points:**

- Rwanda did not reinvent the wheel but worked diligently on implementation of policies known to work in other countries, making sure that everyone has a face mask and has access to enough soap and water to wash their hands while social distancing. Also, enough PPE has been made available to HCPs. Teamwork has been important, from the president on down to the general population.
- Technology has played a big role, from the use of drones broadcasting messages to the general public to robots used to check on patients. Communication via TV, radio, and social media has been crucial. There is no proven drug against COVID-19, so the best approach has been to make sure that hospitalized patients are able to get continuous positive airway pressure. There has not been a great need for ventilators as of this time.
- For patients with T1D, it is important to minimize the risk of getting infected by making sure they have enough medical supplies. Since the majority have mobile phones, some medical consultations have been made over the phone. It is important to have their diabetes controlled. Insulin and other needs are imported, which requires the operation of international flights. So far, there have not been any major interruptions in flights.

**Kayo Waki, MD, MPH, PhD**

The University of Tokyo Graduate School of Medicine, Tokyo, Japan

**Key Points:**

- The guiding principle behind most COVID-19 policies in Japan is avoidance of “Three Cs”—closed spaces with poor ventilation, crowded places with many people nearby, and close-contact settings such as close-range conversations—promoted by the Ministry of Health, Labor and Welfare. Our hospital staff and patients wear masks, measure their body temperature twice daily, and use antiseptic on their hands. During the first wave, hospitals postponed all nonessential elective surgeries, such as outpatient clinical activity, so that remote medical services were promoted. Now, hospital services are getting back to normal, but medical tourism services and consultations for international patients are still postponed. In addition, we conduct PCR tests for COVID-19 for all patients who are going to have surgery at the hospital to avoid and detect in-hospital infection. So far, none of our staff, including medical professionals at the hospital, has contracted COVID-19 because of the high compliance with hospital guidelines. All staff members are not allowed to visit crowded places or dine with non-household groups in or out of the hospital.
- EUAs are expected to be implemented in Japan; so far, remdesivir has received fast-track approval, but there are several pre-conditions necessary for fast-track approval. To improve our ability to act quickly against a pandemic in the future, we should implement an EUA protocol as soon as possible. In addition, a case reporting system is expected to be integrated in Japan. At present, it is not digitized in most parts of the country. Most institutions in local cities and rural areas depend on manual procedures, which delay the identification of new cases.
- Comprehensive online medical care policies for patients with diabetes will keep them safe and offer long-term, consistently available treatment. Online medical care is approved as a special measure during the pandemic, so its use has thus far been relatively limited. Only 20% of all hospitals have implemented this type of care. It is expected that more hospitals will eventually introduce telehealth, increasing accessibility to care.

### Summary of Panel

**Figure 13.**
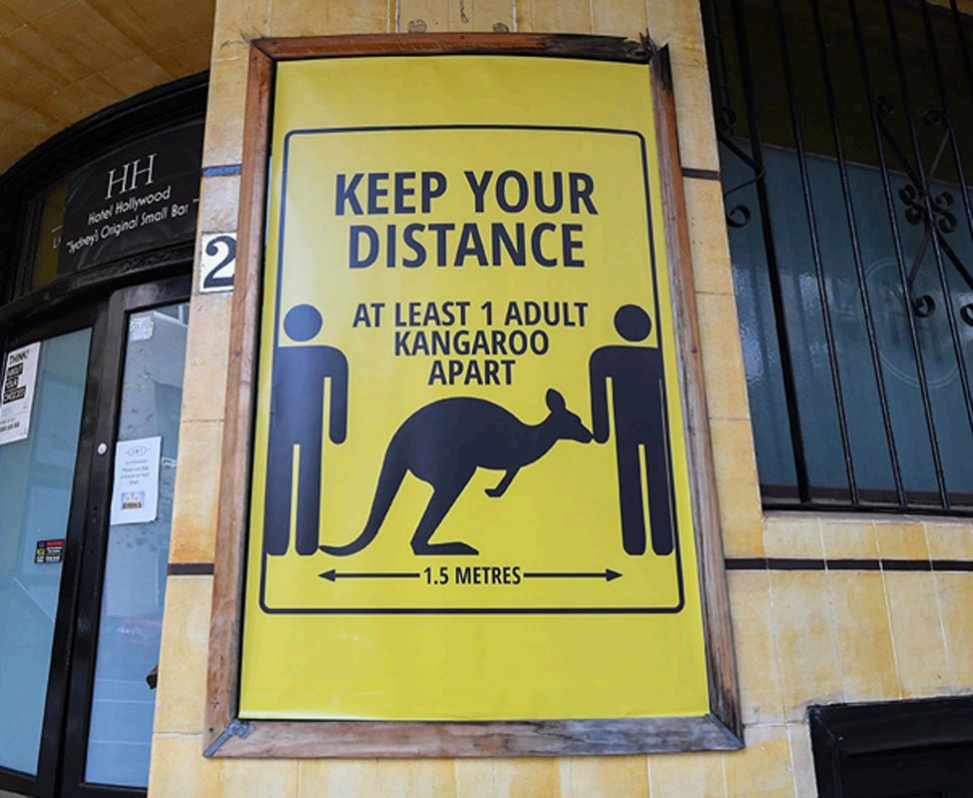
A sign in Sydney, Australia, asking people to socially distance from others. Image from James D. Morgan, Getty Images.^75^

**Figure 14.**
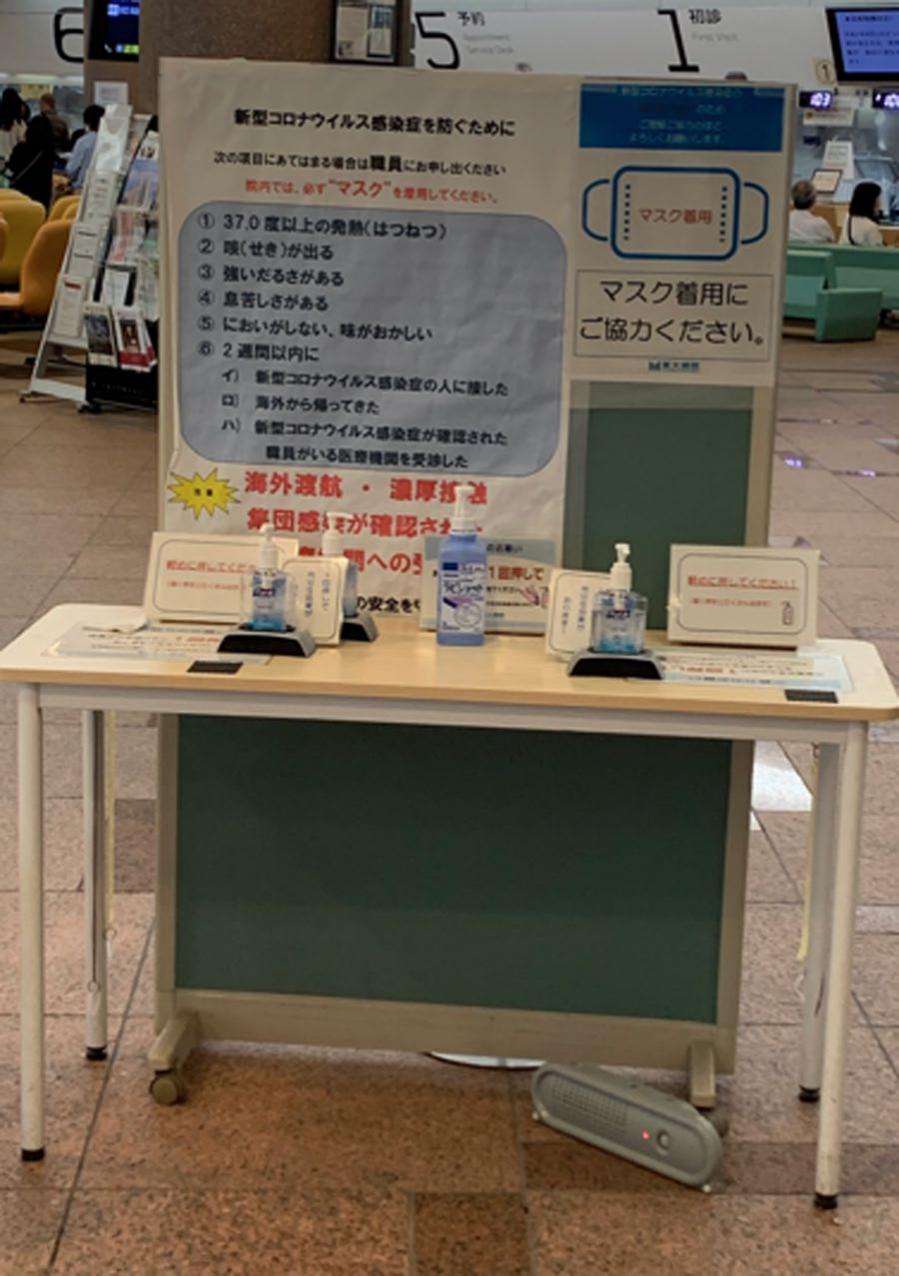
Sanitizers for disinfection that are located at the entrance to a hospital in Tokyo, Japan. All patients are asked to use them when they enter and leave the hospital. Image provided by Kayo Waki, MD, MPH, PhD, The University of Tokyo.

**Figure 15.**
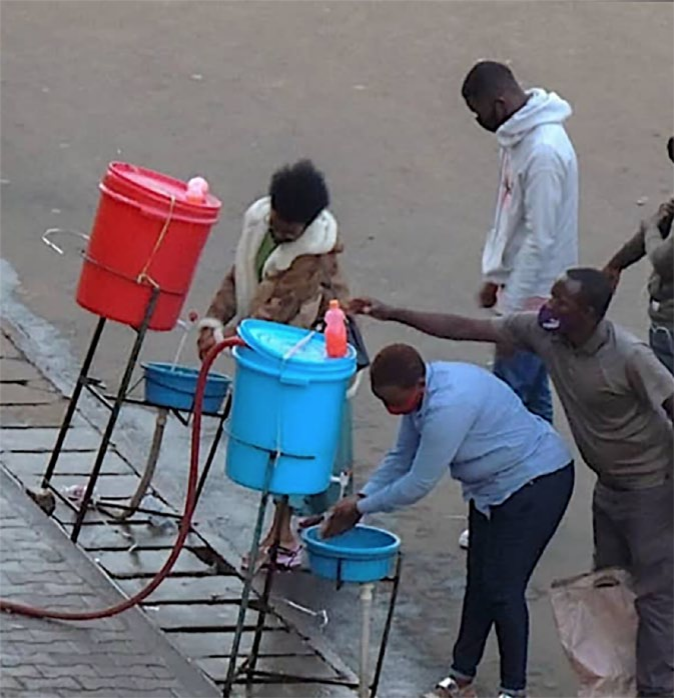
People in Rwanda using outdoor hand-washing stations. Image provided by Laurien Sibomana, MS, Rwandan Diabetes Association.

Endocrinologists, diabetologists, and public health officials from six countries in five continents highlighted the critical needs to follow centralized hospital policies such as isolation, quarantine, and testing for infection control. They also emphasized the importance of social distancing (Figure 13), mask wearing, and hand hygiene (Figure 14) for prevention of virus spread. Patients need speedy access to diabetes advice to (1) prevent admission to the hospital, (2) receive access to diabetes specialist care while being an inpatient, and (3) plan good supportive care after discharge to prevent readmission. Glycemic control during quarantine/isolation can be challenging, and devices such as CGMs and insulin pumps can be useful to remotely monitor and treat patients to protect them and healthcare workers. For countries with limited resources in remote regions and for socioeconomically disadvantaged populations, additional measures are needed to deliver medications, supplies, communications, and clean water for handwashing (Figure 15).

### Section 20: Government Policy

**Ronald Goodstein, PhD**

Georgetown University, Washington, DC

**Key Points:**

- The government, CDC, and medical personnel need to be communicating benefits rather than details.
- People are more sensitive to negative information than they are to positive information. Labeling and alleviating risk will drive behavior.
- In a crisis, there needs to be a single point of communication from the “authority” in charge; all messages must be coordinated and delivered on the same positioning point(s). This has been a mass failure during the current pandemic.

**K. M. Venkat Narayan, MD, MSc, MBA**

Emory University, Atlanta, Georgia, USA

**Key Points:**

- The USA is 4% of the world’s population but has contributed 25% of the cases and deaths from COVID-19. A recent survey rates the government response in the USA as among the worst worldwide.
- The pandemic has shone a torch on many underlying systemic issues relevant to policy, including: (1) lack of consistency between Federal, State, and local policies; (2) socioeconomic and race/ethnic disparities; (3) high prevalence of obesity, diabetes, HTN, and their roles in COVID-19 complications; (4) challenges with healthcare access and affordability; (5) lack of social protection safety nets; (6) high costs of healthcare; (7) inequitable access to technology; and (8) lack of well-coordinated and well-resourced national surveillance and public health strategies and plans.
- As a nation, going forward, we need to rethink a number of critical systems to: (1) implement better and more nimble national surveillance systems, (2) design better national public health response systems, (3) resource a public health workforce, (4) stay attentive to socioeconomic disparities in health, (5) strengthen independent institutions such as CDC and FDA, (6) improve science education of the public, (7) ensure universal accessible healthcare, (8) strengthen primary care, and (9) link technology and social good.

**Nancy Nielsen, MD, PhD**

University at Buffalo, Buffalo, New York, USA

**Key Points:**

- How health services are financed really matters in a pandemic (prospective or population-based pay vs fee-for-service).
- The shortcomings of employer-based health insurance are highlighted with widespread job loss.
- Regulatory flexibility is key, but unintended consequences can result, as seen in widespread nursing home infections.

**Bruce Quinn, MD, PhD**

Bruce Quinn Associates LLC, Los Angeles, California, USA

**Key Points:**

- For patients with diabetes and possible COVID-19, the Centers for Medicare & Medicaid Services (CMS) aggressively created liberal coding, pricing, and coverage guidelines, which will remain in place until the end of the public health emergency.
- Through emergency rulemaking, CMS liberalized virtual meeting rules for diabetes prevention education.
- Overall, CMS has been proactive in liberalizing telehealth rules and appears committed to maintaining some of these expansions.

### Summary of Panel

The USA’s response to COVID-19 has been disappointing and has had many shortfalls, including the inconsistency between Federal, State, and local policies. With a lack of consistent, accurate information from a single reliable source of authority, there has been a general failure in communicating important messages to the public. Furthermore, communication with the public should be focused on benefits rather than details to incentivize people to follow guidelines and safe practices. The pandemic has also highlighted underlying, preexisting issues in healthcare delivery in the USA, including socioeconomic and race disparities, inaccessibility of healthcare services, and shortcomings of employer-based health insurance. As the pandemic situation evolves, it is key to have flexibility in regulation. However, there might be unforeseen risks with overly flexible policy implementation, so foresight and thorough analysis are necessary. Successful policy changes so far have included collaborations at many levels between government agencies, such as CDC, NIH, the FDA, CMS, other public and/or private healthcare agencies and enterprises, caretakers, researchers, and the general public. CMS has covered telehealth and diabetes prevention programs during the pandemic. For the future, we will need to remember the issues that arose during the current pandemic and amend how we handle them both to prepare for a second wave and to improve the general health of the public. Table 5 shows policies and actions during the COVID-19 pandemic that worked at the Federal, State, and regional levels (panel A), plus policies and actions that did not work (panel B) and that really did not work (panel C). Some specific policies that are now needed include: (1) better and more nimble national surveillance systems, (2) more effective nationally coordinated public health response policies, (3) attention and research devoted to addressing the socioeconomic disparities in health, (4) universally accessible healthcare, (5) stronger primary care and independent institutions, (6) unified messages that are focused on individuals as well as the public, and (7) stronger public trust of the government and science.

**Table 5.** The Effectiveness of Policies and Actions During the COVID-19 Pandemic.

**A**	**What worked during the pandemic**		
	**Federal**		Payment for telemedicine services
			EUA for tests and drugs
			Requirement of all insurers to cover testing with no cost sharing and no pre-authorization
			Creation of federal emergency paid sick leave program
	**State**		Special enrollment period for marketplaces
			Waivers for cost-sharing for COVID-19 treatment
	**Regional/Insurer**		Prospective payments, not fee-for-service
**B**	**What did not work**	**C**	**What really did not work**
	Obtainment of ventilators, PPE, and testing materials was like the Wild West		Distribution of supplies, availability of healthcare workers
	Contact tracing was useless when community spread was already high		Reliable testing with acceptable turnaround time
	Early CDC tests were faulty and distribution was problematic		Recognition of asymptomatic spread
	EUA of diagnostics without independent verification of reliability		Consistent messaging as lessons were learned

Tables provided by Nancy Nielsen, MD, PhD, University at Buffalo. Abbreviations: CDC, Centers for Disease Control and Prevention; COVID-19, coronavirus disease 2019; EUA, emergency use authorization; PPE, personal protective equipment.

### Section 21: Regulation of Tests and Treatments

**Alexander Fleming, MD**

Kinexum Services LLC, Harpers Ferry, West Virginia, USA

**Key Points:**

- FDA (and other major regulatory authorities) are reviewing COVID-19-related proposals and submissions at lightning speed.
- FDA review of diabetes therapies has not been interrupted, but many studies have been delayed, suspended, or closed.
- The pandemic has sparked innovation in “pragmatic” trial design and execution, which is likely to extend to many therapeutic areas, including diabetes.

**Alberto Gutierrez, PhD**

NDA Partners LLC, Rochelle, Virginia, USA

**Key Points:**

- In general, the FDA has been flexible in granting EUAs even in consideration of the EUA policies.
- The majority of EUAs granted for testing have been for molecular tests. Only four antigen tests have received EUAs. Recently, a rapid antigen test has also received an EUA.
- We need to increase the amount of testing. There are many pending applications for EUAs, and the FDA has a lot of work ahead of them.

**Yarmela Pavlovic, JD**

Manatt, Phelps & Phillips, LLP, San Francisco, California, USA

**Key Points:**

- FDA has provided a number of COVID-19-specific enforcement discretion policies that should be considered when evaluating regulatory pathways for new digital health and medical technology products.
- In addition to using the EUA process for diagnostic tests, PPE, and ventilators, FDA has also been creative in using it for digital health tools, such as predictive analytic products for acute patient management.
- When EUA and enforcement discretion has not been appropriate for a digital health product, but the product could play an important role in management of the COVID-19 crisis, FDA has been collaborative about development of rapid regulatory strategies, such as informally expediting 501(k) reviews.

### Summary of Panel

Over 800 applications of COVID-19-related diagnostics have been submitted to FDA and over 200 EUAs have been granted to date (mainly for molecular tests and only four antigen tests), which shows the flexibility and speed of FDA to meet the high demands of COVID-19 testing. At the time of the meeting, only two COVID-19 treatments were the subject of EUAs (remdesivir and CP). However, remdesivir was later approved for use in adult and pediatric patients who are over 12 years of age and weighing at least 40 kilograms on October 22, 2020.^76^ The FDA has also formulated a number of COVID-19-specific enforcement discretion policies for new digital health and medical technology products, such as predictive analytic products for acute patient management. Moreover, FDA has been collaborative about developing rapid regulatory strategies, such as informally expediting 510(k) reviews. The agency has sparked innovation in “pragmatic” trial design and execution, which is likely to extend to many therapeutic areas, including diabetes. Serious challenges ahead include the interruptions of clinical trials and scientific research by the pandemic, as well as the increasing need for testing that is fast and inexpensive. Going forward, it is important to also consider the successes and lessons learned from previous experiences with hydroxychloroquine and inaccurate tests.

### Part H: The Future

Moderator: Gerard Coté, PhD

Texas A&M Engineering Experiment Station Center for Remote Health Technologies and Systems, Department of Biomedical Engineering, Texas A&M University, College Station, Texas, USA

#### Section 22: Digital Health Technology

**Jeffrey Joseph, DO**

Thomas Jefferson University, Philadelphia, Pennsylvania, USA

**Key Points:**

- The wearable Trachea Sound Sensor will accurately and continuously monitor a patient’s heart rate, respiratory rate, tidal volume, breathing pattern, oxygen saturation, temperature, body position, and activity level.
- The diagnostic algorithm will use deep machine learning methods to recognize subtle changes in a patient’s cardiorespiratory function to diagnose a COVID-19 viral infection prior to overt symptoms.
- The diagnostic algorithm will use clinical knowledge and deep machine learning methods to calculate a risk-index-score with alerts and alarms for worsening pulmonary function due to a COVID-19 or influenza viral infection.

**Jessie Juusola, PhD**

Evidation Health, San Mateo, California, USA

**Key Points:**

- Digital health tools, including consumer grade (as opposed to clinical grade), offer valuable opportunities to meet people where they are at and understand their real-world health experience with risk factors for COVID-19 (eg, diabetes) or with COVID-19 itself in ways that were previously not possible.
- We can use tools to connect with people remotely and bring in novel data streams. Through those streams, we can run studies and observe people to understand what is working and what is not working, and how much burden is being experienced.
- Being able to measure diabetes burden as well as COVID-19 symptom severity will allow us to better understand, forecast, and affect the health economic impact of COVID-19.

**Bill Evans, BA**

Rock Health, San Francisco, California, USA

**Key Points:**

- The COVID-19-driven shift to telehealth represents an acceleration of adoption of transactional telecare. The real impact will be felt as consumer preference and payment systems shift to favor remote care paradigms in response, driving adoption of higher-complexity higher-value care into remote models.
- Private market investors have shifted dollars away from digital “fitness and wellness” and toward tech-enabled “on-demand healthcare services.” The extent to which this shift is durable beyond the pandemic will be a function of the sustainability of pandemic-driven healthcare economics and regulatory loosening. Though I do not expect this, if capital dries up or “flinches” on the tail end of the pandemic, then we could see a pullback.
- Strategic investors—healthcare and life science companies—have traditionally played an outsized role (relative to other industries) in funding the digital startup ecosystem. Their share of private investment dollars has increased in the first half of 2020, much to our surprise. This either means that “smart money” is leaning into digital transformation, or the herd is moving together. Rock Health has a view on this, but time will tell.

#### Summary of Panel

**Figure 16.**
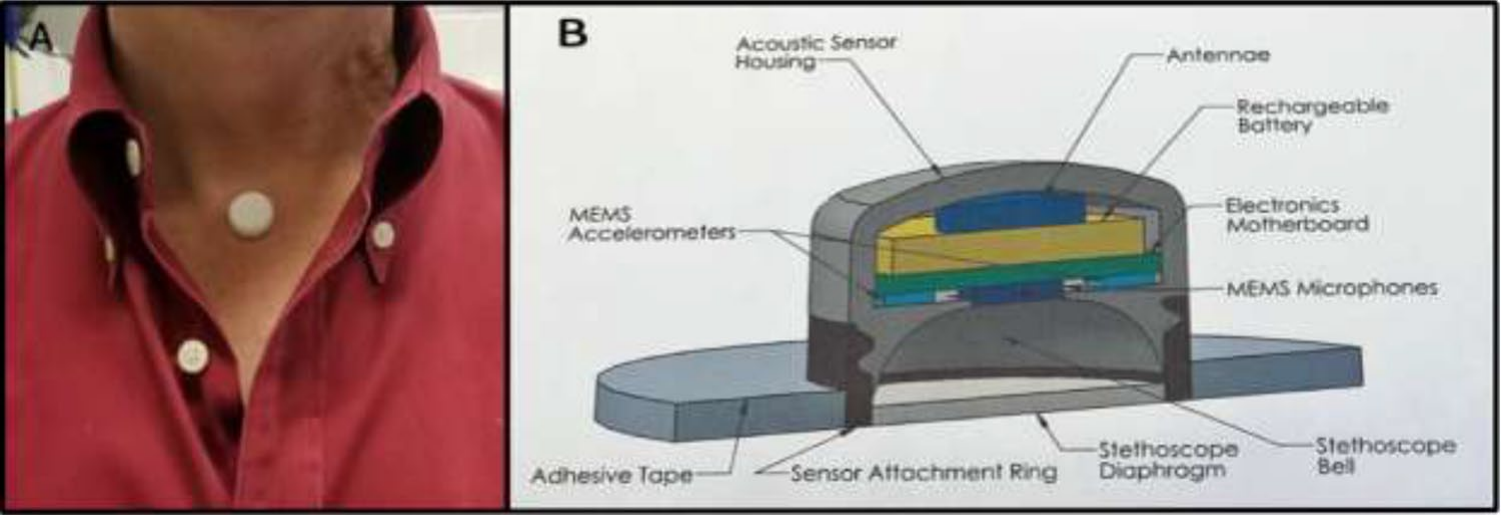
(a) A wearable trachea sound sensor attached to the throat. (b) A diagram of the internal and external components of the trachea sensor. The wearable trachea sensor and related smartphone software application are being developed by RTM Vital Signs, LLC (Philadelphia, PA) in collaboration with Thomas Jefferson University. Image and figure provided by Jeffrey Joseph, DO, Thomas Jefferson University.

Digital health is generally thought of as the use of communication technology to help improve the health and wellness of a patient. The panel included an anesthesiologist clinician/entrepreneur, an executive director of a digital health outcomes company, and the CEO of a healthcare investing venture fund. Together, they brought a diverse perspective on various aspects of digital health. Digital health technologies have been transitioning over the past decade from intermittent vital sign measurements by several devices and infrequent Skype visits to more continuous automatic monitoring with more integrated, often wearable, devices and machine learning diagnostic algorithms. For example, a wearable trachea sound sensor (Figure 16) coupled to a cell phone diagnostic might help with diagnosing abnormal breathing patterns consistent with COVID-19 before the patient is symptomatic. Digital health technologies could be used to bring digital and virtual health to the next level to help monitor patients remotely, particularly in the post-COVID-19 era. Existing tools can be used for collecting real-world data to enable both population and individual patient health. These data could lead to both understanding the burden of COVID-19 and comorbidities like diabetes as well as to supporting clinical interventions. Finally, the view of the market around digital health was discussed with rather large recent investment trends in the areas of on-demand healthcare services, remote monitoring of disease (similar to the wearable trachea sound sensor), and digital therapeutics. Strategic investors from major corporations are now the primary investors in digital health, which is somewhat counter to what one would expect in a downturn, but this was hypothesized to have occurred because these investors better understand the opportunities in digital health.

#### Section 23: Big Data Statistics

**Christina M. Astley, MD, ScD**

Boston Children’s Hospital, Harvard University, Boston, Massachusetts, USA

**Key Points:**

- Big data is timely and nimble, which are critical features during a pandemic.
- Big data tools should strive to enable improved care and inform public health measures.
- It is important to foster and integrate collaborative efforts in the design, analysis, and interpretation of big data to allow for replication, assess for bias, and ensure generalizability across populations, space, and time.

**Bobak Mortazavi, PhD**

Texas A&M University, College Station, Texas, USA

**Key Points:**

- It is important to understand how to leverage time-varying techniques and modify models.
- Collaborative transparent analyses that can be replicated and can be developed through team science are the future of diabetes tech research.
- The pandemic has propelled diabetes technology research and applications for clinical care forward—the future is now.

#### Summary of Panel

**Figure 17.**
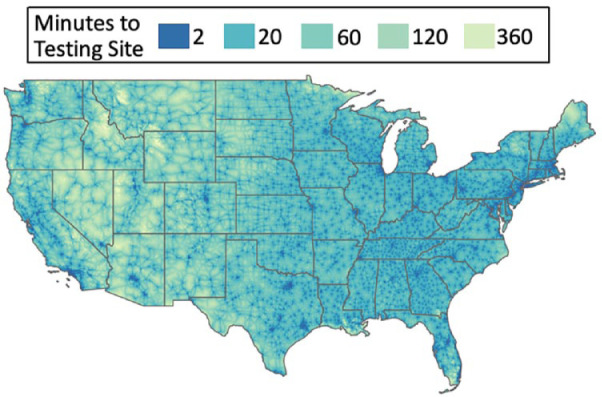
A map of geographic access as measured by the shortest travel time from a 1 km^2^ region to the nearest SARS-CoV-2 testing site in the USA as of May 2020. Methods as per Rader et al,^80^ 2020 *Journal of Travel Medicine.* A total of 6236 testing sites were identified and used to generate this map. Figure provided by Christina M. Astley, MD, ScD, Boston Children’s Hospital, Harvard University and Benjamin Rader, MPH, Boston Children’s Hospital, Boston University.

Big data statistics were covered in this panel from the diverse perspectives of both a medical doctor experienced in translational research and computational epidemiology and a computer scientist working in the area of remote digital health. The focus was on three themes including: (1) big data as a timely and nimble topic, (2) collaborative and replicable analysis, and (3) the future of diabetes technology and data. Highlights were presented in the areas of (1) big data collection regarding disparities in healthcare, (2) differential health-seeking behavior, and (3) disease prediction with a focus on diabetes and COVID-19. A theme involving all three areas was how to provide pandemic care for patients in underserved and underrepresented populations that may have less access to COVID-19 testing. Geographic healthcare disparities are illustrated in Figure 17, which is a map of the shortest travel time from a 1 km^2^ region to a to SARS-CoV-2 testing site. In one particular example, data from Massachusetts children with diabetes who use CGMs have revealed that COVID-19 has impacted their percentage of time in target glycemic range (Christina M. Astley: unpublished data). Specifically, their time out-of-range has decreased since schools were closed in the spring of 2020, suggesting the stay-at-home protocol actually provided a better opportunity for the children to stay in range. Similar data have been reported from Spain^77^ and Italy^78^; however, a study from Israel showed that glycemic metrics for children who use CGMs were similar before and after lockdown started.^79^ The panel also highlighted that big data from digital health devices is only useful if you can use it to impact care. It was noted that data like biomarker trajectories were useful to define and cluster different cohorts and that time-varying statistics and contextual awareness were helpful in developing end-to-end nimble solutions.

#### Section 24: Patient Surveillance and Privacy

**David A. Drew, PhD**

Massachusetts General Hospital, Harvard Medical School, Boston, Massachusetts, USA

**Key Points:**

**Figure 18.**
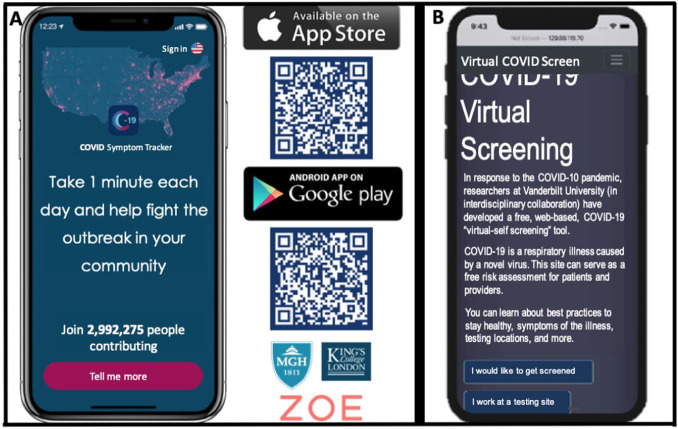
Mobile applications designed to screen for COVID-19. (a) The COVID-19 Symptom Study mobile app on a smartphone. To the right of the smartphone, QR codes are included that allow scanners to download the application through their appropriate application stores. This project, designed to capture COVID-19 symptoms and health patterns in the general public, was developed as an academic-industry collaboration between Massachusetts General Hospital, King’s College London, and ZOE Global Limited.^81^ Figure provided by David A. Drew, PhD, Massachusetts General Hospital, Harvard Medical School. (b) Vanderbilt’s mobile platform for virtual COVID-19 screening. This screening tool was created through collaboration between T.S. Harvey and Thomas Scherr from Vanderbilt University.^82^ Figure provided by T.S. Harvey, PhD, Vanderbilt University. Abbreviation: COVID-19: coronavirus diseases 2019.

- The COVID-19 Symptom Study mobile phone application empowers real-time epidemiology methods guided by and using principles of informed consent for research studies. (Figure 18[a])

- Symptom-based zip code level location data is sufficient to identify regional hotspots in advance of public health reporting of tests.
- While mobile phone-based survey applications empower real-time data collection, there are limitations associated with access and study participation, which introduce bias that must be controlled and acknowledged.

**Elissa Weitzman, ScD, MSc**

Boston Children’s Hospital, Harvard University, Boston, Massachusetts, USA

**Key Points:**

- Privacy is a key driver of well-being and a patient safety concern. There is good evidence that both the COVID-19 pandemic and diabetes can be advanced through surveillance that uses online and personally generated data from apps and digital health tools. Concerted attention is needed to mitigate risks to privacy from poorly developed and inconsistently applied protections, or we risk alienating and even harming the populations from which data derive. Special consideration is needed for pediatric populations for whom existing privacy protections are lax/missing, and for members of demographic groups at risk from healthcare and social disparities. Risks may be especially acute in the USA and other societies where harm to citizens’ abilities to obtain insurance, healthcare, employment, and housing may arise from disclosure of information.
- Privacy is an international commodity. Global solutions and collaborations may be helpful to developing an actionable framework for supporting privacy, given differences in privacy protections and norms across countries and economies. This inconsistency is especially so for controlling COVID-19, where real time surveillance over time and space is vital to curtailing disease spread. Addressing barriers related to fundamental differences in beliefs about the importance of privacy, commitment to its protection, and values for balancing technical and business innovation with citizen protections need to be addressed. Understanding the sociology of these issues and fostering robust collaborations and shared understanding is as, if not more, important than understanding the technologies.
- Patient and population (public) health are both vitally important, and sacrificing one for the other is a problematic tradeoff. A “one-size-fits-all” approach for online systems that support COVID-19 or diabetes surveillance may not be practical. However, policy and technological goals for privacy need to align around optimizing privacy protections for both individual patient and population health needs. Transparency around data use is a key and measurable indicator of privacy—clarity around how data are shared and used in the digital marketplace may be the most important safeguard to protecting privacy and balancing concerns for patient and population health protections.

**T.S. Harvey, PhD**

Vanderbilt University, Nashville, Tennessee, USA

**Key Points:**

- Interdisciplinary developments: The development of virtual-self screening tools to promptly identify, map, and reduce the public health risk of infectious diseases like COVID-19 and their impacts must be interdisciplinary efforts that emerge at the intersection of technology, public health, and social science research.
- Public-centered designs: If the intent is that virtual-self screening instruments, like Vanderbilt’s COVID-19 tool (Figure 18[b]) are to be utilized by the “general” public, then designs beyond incorporating the latest and most accurate public health guidelines must also reflect and integrate (from end-to-end) research on public perceptions of risk. These perceptions (within target populations) are critical to the public’s uptake of the tool.
- Politics and public health in the time of a pandemic: Technological innovations like virtual self-screening tools, no matter how thoughtfully designed or how widely circulated they are, do not exist outside of the wider context of the political, historical, cultural, linguistic, and economic environments in which they are deployed. Moving forward, development teams must consider, navigate, and even anticipate these factors as a part of their deployment design if the intended populations, research, and public health objectives are to be reached.

**Dave Kleidermacher, BS**

Google, Mountain View, California, USA

**Key Points:**

- Privacy and user/patient trust are at the heart of the Google/Apple design for the COVID-19 mobile device exposure notification system.
- Common theme between DTSec/IEEE P2621 (diabetes device security/privacy standard) and COVID-19 exposure notification system: transparency is the key to building trust in privacy.
- Balancing patient health/safety and digital privacy is difficult—sometimes they are at odds—and we need more cross-stakeholder alignment on principles to help guide industry, regulators, patients, caregivers, and so on.

#### Summary of Panel

The panel discussed patient surveillance using mobile applications, also known as apps, and the importance of building trustworthy apps that protect the privacy of their users. The first app discussed was the COVID-19 Symptom Study mobile app (Figure 18(a)), which provides real-time pandemic epidemiology. This smartphone app does not have contact tracing, which would involve tracking patients who have been exposed and/or who have tested positive for COVID-19 as well as warning those who have been exposed to individuals who may be carrying the virus.^83^ There are no passive data collected from the users—the only data that are collected are what each participant voluntarily provides. Participants are made aware of the data that will be collected. The app was designed to be easy to use and not time-consuming, with the patient describing how they are feeling. It provided early real-time insight into COVID-19 epidemiology with identification of hot zones using provided zip code data. However, this mobile app has had limitations because of sampling and selection bias and study access.^84^ Additionally, patient surveillance must account for social science aspects, including (1) how can the technology be trusted (because some of the most vulnerable are also the most suspicious), (2) who would be using the tool, (3) how the questions are asked, and (4) what is the sequence of the questions. A mobile tool is not valuable if it is not used, so it is important to make the app trustworthy and accessible. A mobile platform for virtual COVID-19 screening developed at Vanderbilt University (Figure 18[b]) was designed with all of these social science considerations in mind. It is also critical to consider and understand the public perception of risk to develop an app that will be well-received and widely used. When designing patient surveillance tools, it is important to understand the pandemic as a developing situation, rather than a single event, to ensure that the tool will stay relevant and continue to be used by the intended populations. Finally, security exposure notifications were discussed in the context of Google and Apple working together to design a system that alerts people if they were exposed to an individual that was COVID-19-positive. To protect the users’ privacy, the system must be trustworthy in terms of putting privacy first and cannot ask for locations or identifying information from the individuals who use the system. Informed consent and transparency is of utmost importance when it comes to building trust between the user and the app.

Privacy is a key driver of well-being and a patient safety concern (particularly for children for whom regulatory controls governing privacy are poorly developed). Disclosure/breach of privacy has the potential to create harm and widen health disparities. For example, disclosure of preexisting health conditions can place patients at a disadvantage for employment, housing, life insurance, and other opportunities. It may exacerbate stigma. The language of privacy protections and terms of use can be inscrutable, making it especially hard for persons with lower levels of education to understand them, and contributing to inequalities. Privacy is an international commodity—insofar as regulatory standards for protecting health and personally identifiable information including in the setting of apps and digital health tools vary across countries and global regions. This inconsistency can create barriers to international cooperation. Both the competitiveness of businesses and the capacity of health surveillance efforts are affected by differences in standards for protecting privacy – these differences have created potential for conflict and competing interests or a “trade war” and “standards war” that centers on privacy. Such conflict erodes the level of cooperation needed to handle health threats, a problem that is especially acute in the setting of a global pandemic. Some formulations of privacy prioritize the wishes and needs of the patient, while others place emphasis on decision making that centers on the need to know and value of information sharing with respect to population health. At the end of the day, both are essential, not just one or the other and effective privacy protections will need to balance concern for these issues within a system.

## Conclusion

This conference has highlighted the impressive amount of rapid collaboration, research efforts, and technology advancements focused on the COVID-19 pandemic, in order to support people with diabetes. The purpose of this meeting was to study what we can do to protect patients with diabetes from COVID-19 and how to treat them if they develop this infection.

Seven key themes were discussed by many of the 79 panelists during the summit: (1) diabetes patients are at increased risk of complications from COVID-19, and diabetes patients require protection from and vigorous treatment of COVID-19^85^; (2) further epidemiological study is needed to understand reasons why diabetes confers elevated risk for adverse outcomes from COVID-19, which will require identification of biomarkers and risk factors for morbidity and mortality in the diabetes population^86^; (3) adverse SDoH predict and lead to poor outcomes in both diabetes and COVID-19^87^; 4) telehealth is a new paradigm for treating diabetes in the pandemic era because people are reluctant to travel to a healthcare facility where they are at risk of exposure to COVID-19, and sensor data can now be automatically uploaded for remote asynchronous review (however some procedures cannot be replaced by telehealth at this time)^88^; (5) the use of CGMs, which is becoming widespread for outpatients, has the potential to be imported into the hospital setting for diabetes patients with COVID-19 to improve care, save time, decrease nursing exposure, and preserve PPE^51^; (6) because future pandemics of infections like COVID-19 could affect patients with diabetes particularly adversely, preparation is needed to develop policies for surveillance, data privacy, consistent messaging, contact tracing, mask wearing,^89^ social distancing,^90^ stockpiling of PPE, and allocation of scarce resources; and (7) the COVID-19 pandemic has accelerated development and regulation of numerous digital technologies for remote management of diabetes, including methods of physiological monitoring, data analysis, and communication, which will have positive effects on diabetes management in the future.^91^

In conclusion, the International COVID-19 and Diabetes Summit has illustrated how the COVID-19 pandemic has suddenly resulted in new attitudes toward and practices for healthcare delivery for people with diabetes. The management of diabetes will never be the same.
